# Overexpression of thermostable *meso*-diaminopimelate dehydrogenase to redirect diaminopimelate pathway for increasing L-lysine production in *Escherichia coli*

**DOI:** 10.1038/s41598-018-37974-w

**Published:** 2019-02-20

**Authors:** Jian-Zhong Xu, Hao-Zhe Ruan, Li-Ming Liu, Lu-Ping Wang, Wei-Guo Zhang

**Affiliations:** 1 The Key Laboratory of Industrial Biotechnology, Ministry of Education, School of Biotechnology, Jiangnan University, 1800# Lihu Road, WuXi, 214122 People’s Republic of China; 2 State Key Laboratory of Food Science and Technology, School of Biotechnology, Jiangnan University, 1800# Lihu Road, WuXi, 214122 People’s Republic of China

## Abstract

Dehydrogenase pathway, one of diaminopimelate pathway, is important to the biosynthesis of L-lysine and peptidoglycan via one single reaction catalyzed by *meso*-diaminopimelate dehydrogenase (DapDH). In this study, the thermostable DapDH was introduced into diaminopimelate pathway that increased the final titer (from 71.8 to 119.5 g/L), carbon yield (from 35.3% to 49.1%) and productivity (from 1.80 to 2.99 g/(L∙h)) of L-lysine by LATR12-2*∆rpiB::ddh*_St_ in fed-batch fermentation. To do this, the kinetic properties and the effects of different DapDHs on L-lysine production were investigated, and the results indicated that overexpression of *St*DapDH in LATR12-2 was beneficial to construct an L-lysine producer with good productive performance because it exhibited the best of kinetic characteristics and optimal temperature as well as thermostability in reductive amination. Furthermore, ammonium availability was optimized, and found that 20 g/L of (NH_4_)_2_SO_4_ was the optimal ammonium concentration for improving the efficiency of L-lysine production by LATR12-2*∆rpiB::ddh*_St_. Metabolomics analysis showed that introducing the *St*DapDH significantly enhanced carbon flux into pentose phosphate pathway and L-lysine biosynthetic pathway, thus increasing the levels of NADPH and precursors for L-lysine biosynthesis. This is the first report of a rational modification of diaminopimelate pathway that improves the efficiency of L-lysine production through overexpression of thermostable DapDH in *E. coli*.

## Introduction

**Figure 1:**
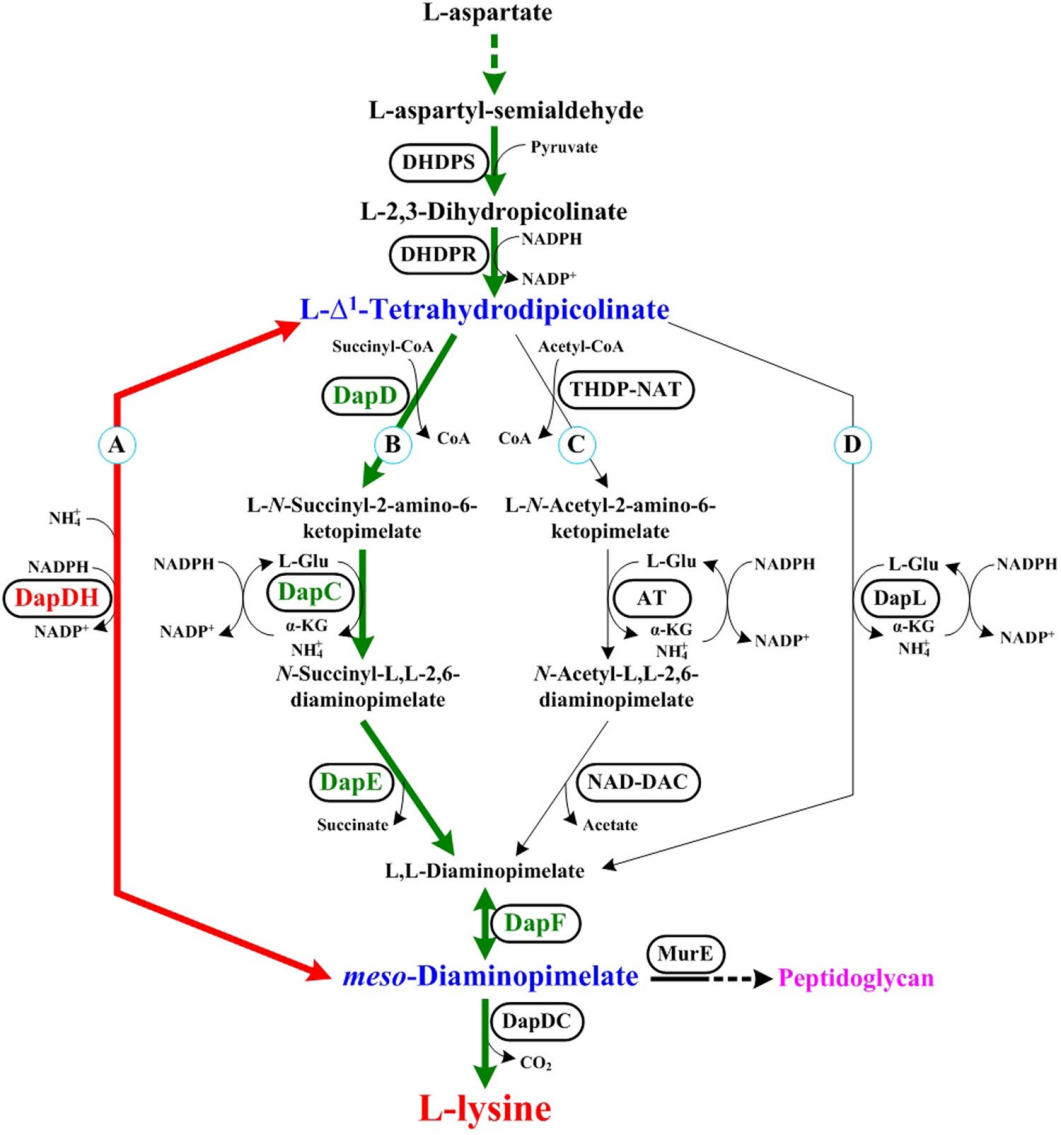
Variant pathways for the synthesis of *meso*-DAP/L-lysine in the prokaryotes, archaea, *Chlamydia* and plants: dehydrogenase pathway, (**A**) succinylase pathway, (**B**) acetylase pathway, (**C**) and aminotransferase pathway. (**D**) *meso*-DAP/L-lysine biosynthetic pathway present in *E. coli* is labeled as green lines. The introduced pathway in *E. coli* is labeled as red lines. Enzymes are listed in the boxes. Abbreviations: *DHDPS* Dihydrodipicolinate synthetase, *DHDPR* Dihydrodipicolinate reductase, *DapDH meso*-Diaminopimelate dehydrogenase, *DapD* Tetrahydrodipicolinate succinylase, *DapC* Succinyl-amino-ketopimelate transaminase, *DapE N*-succinyl-diaminopimelate desuccinylase, *DapF* Diaminopimelate epimerase, *THDP-NAT* Tetrahydrodipicolinate acetylase, *AT N*-acetylaminoketopimelate aminotransferase, *NAD-DAC N*-acetyl-diaminopimelate deacetylase, *DapL* Tetrahydrodipicolinate aminotransferase.

L-lysine, one of the essential amino acids for animals and humans^1^, is widely used in feed, food, and pharmaceutical industry, *etc*. The global marketplace for L-lysine is expected to amount to $6.96 billion by 2020 as consumption increases^2,3^. In industry, L-lysine is mainly produced by microbial fermentation employing mutant strains of bacteria, such as *Corynebacterium* sp. and *Escherichia* sp^4,5^. Therefore, an L-lysine producer with excellent fermentability is needed to increase the final titer and to reduce the production cost. The L-lysine biosynthetic pathway is start from L-aspartate and enters into diaminopimelate (DAP) pathway (Fig. 1)^6^. The DAP pathway starts from L-aspartyl-semialdehyde, and exists four variant pathways in the prokaryotes, archaea, *Chlamydia* and plants: the succinylase, acetylase, dehydrogenase, and aminotransferase pathways^7,8^. The difference among these variant DAP pathways is that how to produce *meso*-DAP from tetrahydrodipicolinate (THDPA)^9^. Note that most prokaryotes appear to preferentially utilize only one of these pathways. For example, *E. coli* only use the succinylase pathway for *meso*-DAP biosynthesis^10^. However, some bacteria use redundant pathways to biosynthesize *meso*-DAP. For example, *C. glutamicum* possess the succinylase and dehydrogenase pathways^11^, and *Bacillus macerans* possess the acetylase and dehydrogenase pathways^12^. In addition, the dehydrogenase and aminotransferase pathways operate in *Clostridium thermocellum* and *Bacteroides fragilis*^9^.

The dehydrogenase pathway converts THDPA to *meso*-DAP in a single step, which is catalyzed by diaminopimelate dehydrogenase (DapDH; encoded by *ddh* gene)^13^. However, the dehydrogenase pathway is only found in a handful of species of bacteria, which is in contrast to the alternative succinylase and acetylase pathways that are the most widely distributed in plants and bacteria^14^. The structure of DapDH has been determined from bacteria, including *C. glutamicum* (*Cg*DapDH)^10,14^, *Ureibacillus thermosphaericus* (*Ut*DapDH)^15^, and *Symbiobacterium thermophilum* (*St*DapDH)^16,17^. These studies have shown that different DapDH has different crystal structure, thereby impacting its performance profile, for example, thermal stability^15^ and substrate affinity^9^. According to the previous reports^9,18^, the dehydrogenase pathway acts as an ancillary pathway for the biosynthesis of L-lysine and peptidoglycan in bacteria. However, it is a prerequisite for the increase of carbon flux to *meso*-DAP^19,20^. In addition, our previous results indicated that it is responsible for the high rate of L-lysine production in *E. coli*^3^. Therefore, introducing or intensifying the dehydrogenase pathway may improve the production performance of the L-lysine producers, thus increasing the carbon yield, final titer and productivity of L-lysine.

*E. coli* is used worldwide for the industrial production of amino acids, including L-lysine^6,21,22^. In *E. coli*, the succinylase pathway is used as the only pathway for *meso*-DAP biosynthesis catalyzed by four enzymatic steps (Fig. 1). Although some studies^3,23^ suggested that introduction of the DapDH from *C. glutamicum* or its subspecies in L-lysine producer *E. coli* was beneficial to increase the L-lysine production, they neglected the differences in the optimal cultivated conditions between *E. coli* and *C. glutamicum*. For example, the temperature optimum for *E. coli* is 37 °C, whereas it is 30 °C for *C. glutamicum*. Note that the activity and stability of the intracellular enzymes in the host cell is changed with different conditions^9,24,25^. In this paper, we introduced a DapDH from different bacteria with different temperature optimum in *E. coli* to investigate its effect on L-lysine production; results indicated that the DapDH from thermophilic bacterium *S. thermophilum* (*St*DapDH) has the positive effects in improving the performance of L-lysine fermentation process by *E. coli* for the first time. In addition, the introducing mode and ammonium availability were also investigated, indicating that the co-existence of two pathways and sufficient ammonium availability are good for increasing the final titer of L-lysine with a high carbon yield and productivity in *E. coli*. These results reported here can serve as a general concept and guidance for breeding high-yielding strains and producing L-lysine in industry.

## Results and Discussions

### Overexpression, purification and function identification of His-tagged DapDH from different bacteria

The DapDH-coding gene *ddh* from different strain shows the huge difference of nucleotide and amino acids sequence identity among these strains (Fig. S1)^9,24,25^. According to previous reports, the DapDH from different strains exhibits different temperature optimum and substrate affinity^9,15^. In order to screen out the best DapDH for L-lysine production in *E. coli*, the six DapDHs from six representative bacteria [including *C. glutamicum* ATCC13032 (Cg2900; *Cg*DapDH), *Bacillus sphaericus* IFO3525 (BAB07799; *Bs*DapDH), *C. therimocellum* ATCC27405 (Cthe_0922; *Ct*DapDH), *B. fragilis* YCH46 (Bf3690; *Bf*DapDH), *S. thermophilum* IAM14863 (Sth1425; *St*DapDH) and *U. thermosphaericus* A1 (AB636161; *Ut*DapDH)] was overexpressed in *E. coli* BL21 (DE3) using pET28a, and then used for investigating their functions and kinetic properties. According to the analysis of SDS-PAGE, the molecular mass of DapDH was about 40 kDa, which was nearly equal to the calculated molecular weights (data not shown). In addition, all of these DapDH orthologs are able to complement the *meso*-DAP auxotrophy of the *E. coli ∆dapD/∆dapE* (Fig. S2), indicating that these DapDHs are the functional forms of DapDH.

The purified enzymes were used for investigating the functions and kinetic properties. All of these DapDHs showed both the activities of oxidative deamination and reductive amination. However, all of these DapDHs catalyzed the reductive amination with higher efficiency than they catalyzed the oxidative deamination except the *Bf*DapDH (Tables 1 and S1). It should be noted that different DapDHs showed a huge difference in the oxidative deamination and reductive amination (Table S1). Although DAP pathway is necessary for cell survival because it involves the peptidoglycan biosynthesis^26^, different bacterial species possess different variants and even different amounts of DAP pathways. For example, *B. sphaericus* only possesses the dehydrogenase pathway^27^, and *C. glutamicum* possesses the dehydrogenase and succinylase pathways^11^, whereas *B. fragilis* and *C. thermocellum* possess the dehydrogenase and aminotransferase pathways^9^. Moreover, the kinetic analysis of these DapDHs again showed that different orthologs had different substrate affinity (*K*_*m*_), thereby affecting the catalytic efficiency of enzyme (Table 1). The *K*_*m*_ of *Bf*DapDH for THDPA (*K*_*m*_ = 0.57 ± 0.14 mmol/L) was nearly five-fold higher than that of *Ct*DapDH (*K*_*m*_ = 0.11 ± 0.03 mmol/L). The kinetic constants were also determined for other DapDHs (including *Cg*DapDH, *Bs*DapDH, *St*DapDH and *Ut*DapDH), indicating that they shared the similar values for THDPA and *meso*-DAP (within the ranges of *Bf*DapDH and *Ct*DapDH), but the kinetic constants towards ${{\rm{NH}}}_{4}^{+}$ of these orthologs were different (Table 1). As can be seen from Table 1, *Bf*DapDH exhibited a lowest *K*_*m*_ for ${{\rm{NH}}}_{4}^{+}$, followed by the *Bs*DapDH, whereas the *V*_*max*_, *K*_*cat*_ and *K*_*cat*_/*K*_*m*_ of *Bf*DapDH were not higher than the others. Although the *Ct*DapDH exhibited a highest *K*_*m*_ for ${{\rm{NH}}}_{4}^{+}$, the *V*_*max*_*, K*_*cat*_ and *K*_*cat*_/*K*_*m*_ were ranked first (Table 1). It is noteworthy that different variants of DAP pathways exhibits the alterable roles on peptidoglycan and L-lysine biosynthesis in different strains under different cultural conditions^28^. All of these factors have contributed to the different activities of DapDH in different strains.

**Table 1:** The kinetic parameter (±SD) of different DapDH for different substrate with NADPH or NADP^+^ as cofactor^a^.

Enzymes	Cofactor	Reaction	Substrate	*V*_*max*_ (U/mg)	*K*_*m*_ (mmol/L)	*K*_*cat*_ (/s)	*K*_*cat*_/*K*_*m*_
*Cg*DapDH	NADPH	Amination	THDPA	2.1 ± 0.3	0.27 ± 0.02	41.9 ± 4.4	155.2
			${{\rm{NH}}}_{4}^{+}$	38.4 ± 3.4	39.2 ± 6.5	768.3 ± 62.5	23.8
	NADP^+^	Deamination	*meso*-DAP	5.8 ± 0.6	2.8 ± 0.3	115.4 ± 15.8	41.2
*Bs*DapDH	NADPH	Amination	THDPA	3.2 ± 0.2	0.23 ± 0.05	64.7 ± 7.3	281.3
			${{\rm{NH}}}_{4}^{+}$	25.6 ± 2.3	10.8 ± 1.02	511.9 ± 27.6	47.4
	NADP^+^	Deamination	*meso*-DAP	7.7 ± 0.5	2.4 ± 0.1	154.2 ± 0.4	64.3
*Ct*DapDH	NADPH	Amination	THDPA	13.9 ± 0.3	0.11 ± 0.03	278.2 ± 25.3	2318.3
			${{\rm{NH}}}_{4}^{+}$	90.8 ± 10.1	89 ± 14.2	1815.6 ± 125.3	205.4
	NADP^+^	Deamination	*meso*-DAP	17.2 ± 1.3	0.21 ± 0.05	344.3 ± 31.4	1638.6
*St*DapDH	NADPH	Amination	THDPA	2.7 ± 0.1	0.24 ± 0.3	52.8 ± 4.5	220.0
			${{\rm{NH}}}_{4}^{+}$	19.4 ± 2.6	6.3 ± 0.7	387.4 ± 45.3	61.5
	NADP^+^	Deamination	*meso*-DAP	7.3 ± 0.5	1.8 ± 0.08	145.7 ± 1.4	80.9
*Bf*DapDH	NADPH	Amination	THDPA	0.24 ± 0.03	0.57 ± 0.14	4.9 ± 0.3	8.6
			${{\rm{NH}}}_{4}^{+}$	8.04 ± 0.96	4.2 ± 0.5	160.8 ± 21.2	38.3
	NADP^+^	Deamination	*meso*-DAP	0.39 ± 0.05	0.11 ± 0.02	7.7 ± 0.4	70.0
*Ut*DapDH	NADPH	Amination	THDPA	2.5 ± 0.1	0.26 ± 0.3	50.6 ± 4.5	194.6
			${{\rm{NH}}}_{4}^{+}$	17.3 ± 2.2	6.5 ± 0.5	347.7 ± 25.8	53.4
	NADP^+^	Deamination	*meso*-DAP	6.2 ± 0.4	1.9 ± 0.2	123.3 ± 9.4	64.9

^a^The mixture for measuring NADPH oxidation contained 200 mmol/L Na_2_CO_3_-NaHCO_3_ (pH 8.5), 0.5 mmol/L NADPH, 200 mmol/L NH_4_Cl, 5.0 mmol/L THDPA and 25 μg of pure recombinant DapDH. The production of NADP^+^ was monitored continuously at A340. The mixture for measuring NADP^+^ reduction contained 200 mmol/L Na_2_CO_3_-NaHCO_3_ (pH 10.0), 5 mmol/L *meso*-DAP, 0.5 mmol/L NADP^+^ and 25 μg of pure recombinant DapDH. The production of NADPH was monitored continuously at A340. The kinetic parameters were determined by varying substrate concentrations while keeping the co-substrate level constant at the set concentration. All assays were carried out at 30 °C.

All data are meaning values of three determinations of three independent experiments with ±SD.

**Figure 2:**
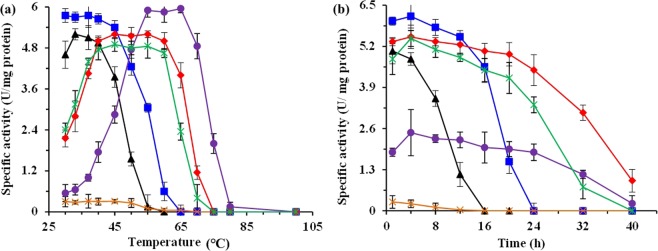
Temperature optimum (**a**) and thermostability under 40 °C (**b**) of different DapDHs from different strains in shake-flasks culture. Signal denotes: *Cg*DapDH (triangle, black), *Bs*DapDH (squares, blue), *Ct*DapDH (circle, purple), *St*DapDH (diamond, red), *Ut*DapDH (asterisk, green), and *Bf*DapDH (cross, orange). The data represent mean values and standard deviations obtained from three independent experiments.

The effect of temperature on the reductive amination of THDPA was determined by assessing the enzyme activity at various incubation temperatures for 1 h. Consistent with the previous results^9,24,25^, the DapDH from thermophiles shows the higher temperature optimum than that from enteric and soil species (Fig. 2a). For example, the purified *Ct*DapDH exhibited a temperature optimum at 65 °C for reductive amination, whereas the temperature optimum was 33 °C for *Cg*DapDH. For *St*DapDH and *Ut*DapDH from thermophiles, the activity was stable over the temperature range of 40 °C to 60 °C and maintained at the high level (Fig. 2a). In addition, the effect of incubation time at 40 °C on the activity of the different DapDHs was also investigated. As shown in Fig. 2b, the activity of all DapDHs was decreased with increase of the incubation time, especially for *Cg*DapDH and *Bs*DapDH. Although the *Ct*DapDH remained stable when incubated at 40 °C for 24 h, it exhibited the relatively low activity as compared with *St*DapDH and *Ut*DapDH (Fig. 2b).

### Inhibition of different DapDHs on reductive amination by nucleotide-cofactor, substrate and product

**Figure 3:**
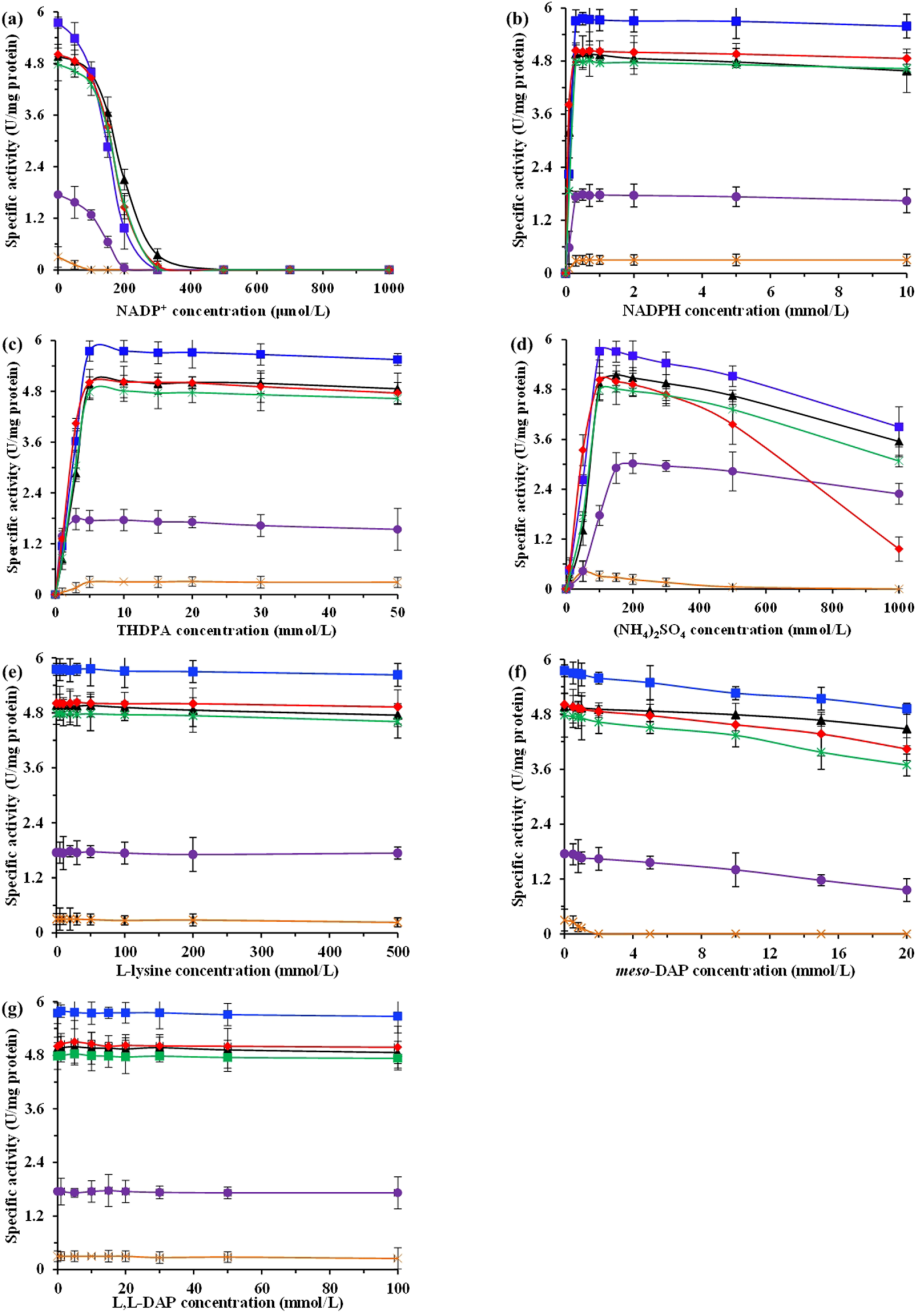
Inhibition of different DapDHs on reductive amination by nucleotide-cofactor, substrate and product in different assay mixture at temperature of 40 °C, that is, with NADP^+^ as the variable parameter (**a**), with NADPH as the variable parameter (**b**), with ${{\rm{NH}}}_{4}^{+}$ as the variable parameter (**c**), with THDPA as the variable parameter (**d**), with *meso*-DAP as the variable parameter (**e)**, with L,L-DAP as the variable parameter **(f**), and with L-lysine as the variable parameter (**g)**, respectively. Signal denotes: *Cg*DapDH (triangle, black), *Bs*DapDH (squares, blue), *Ct*DapDH (circle, purple), *St*DapDH (diamond, red), *Ut*DapDH (asterisk, green), and *Bf*DapDH (cross, orange). Each data point was measured in duplicate or triplicate, and error bars show the standard deviation.

DapDH is a bifunctional enzyme catalyzing the NADPH-dependent reductive amination to form *meso*-DAP with THDPA and ${{\rm{NH}}}_{4}^{+}$ as substrates and the NADP^+^-dependent oxidative deamination to form THDPA with *meso*-DAP as substrate^29^. In order to investigate whether nucleotide-cofactor, substrate and product involved in DapDH-catalyzed reaction regulate the activity of DapDH, the effects of nucleotide-cofactor, substrate and product on different DapDHs were studied on the reductive amination. For all of these DapDHs, the nucleotide-cofactor NADP^+^ showed the competitive inhibition with NADPH in the presence of a high as well as constant THDPA and ${{\rm{NH}}}_{4}^{+}$ concentration, whereas it showed the noncompetitive inhibition with THDPA or ${{\rm{NH}}}_{4}^{+}$ in the presence of a high as well as constant NADPH and ${{\rm{NH}}}_{4}^{+}$ or THDPA concentration (Fig. S3). This is because DapDH is a bifunctional enzyme catalyzing the NADPH-dependent reductive amination and the NADP^+^-dependent oxidative deamination^29^, thus both NADP^+^ and NADPH can be combined with the free form of DapDH^18^. However, the strength of inhibition on different DapDHs presented certain discrepancies (Fig. 3a). For example, the activity of *Ct*DapDH and *Bs*DapDH was dramatically decreased with the increase of the concentration of NADP^+^ (*K*_*i*_ = 7.3 ± 0.6 μmol/L and *K*_*i*_ = 5.8 ± 0.3 μmol/L, respectively), whereas the *Cg*DapDH showed the minimal changes (*K*_*i*_ = 15.2 ± 1.3 μmol/L). The other nucleotide-cofactor NADPH was also tested for its regulating properties. As can be seen from Fig. 3b, no inhibition of these DapDHs was observed at high concentration of NADPH (up to10 mmol/L) with constant THDPA and ${{\rm{NH}}}_{4}^{+}$ concentration.

THDPA and ${{\rm{NH}}}_{4}^{+}$ are the substrates for DapDH in catalyzing reductive amination. To determine the effect of THDPA on DapDHs, assays were performed by varying the concentration of THDPA with constant NADPH and ${{\rm{NH}}}_{4}^{+}$ concentration. In addition, the effect of ${{\rm{NH}}}_{4}^{+}$ was also tested. The results are listed in Fig. 3c,d. No inhibition of these DapDHs was observed at high concentration of the THDPA (up to 50 mmol/L; Fig. 3c). However, the activity of these DapDHs were firstly increased and then decreased with increasing (NH_4_)_2_SO_4_ (Fig. 3d). Especially for *St*DapDH, the activity was dramatically decreased and get closer to 20% of initial at 1 mol/L of (NH_4_)_2_SO_4_ when the concentration of (NH_4_)_2_SO_4_ was above 0.5 mol/L. It is well known that (NH_4_)_2_SO_4_ is a physiologically acid salt^30^. Therefore, excessive concentrations of (NH_4_)_2_SO_4_ changes the pH in the reaction system, thereby missing the optimal pH of DapDH.

Kinetic studies were also carried out to test the products in the L-lysine biosynthetic pathway for inhibition of DapDHs, for example L-lysine, *meso*-DAP and L-isomer of DAP (i.e., *L,L*-DAP). Although L-lysine, as the end-product in pathway, controls multiple enzymes activity, including AK and DHDPS^3,31^, it has no inhibition on oxidative deamination and reductive amination (Fig. 3e). Consistent with the previous results^18,24,32^, the *L,L*-DAP inhibited only the deamination of *meso*-DAP. Conversely, *meso*-DAP inhibited slightly the amination of THDPA, especially for *Bf*DapDH and *Ct*DapDH (Fig. 3f).

### Comparing the effects of the different DapDHs on L-lysine production in *E. coli*

**Figure 4:**
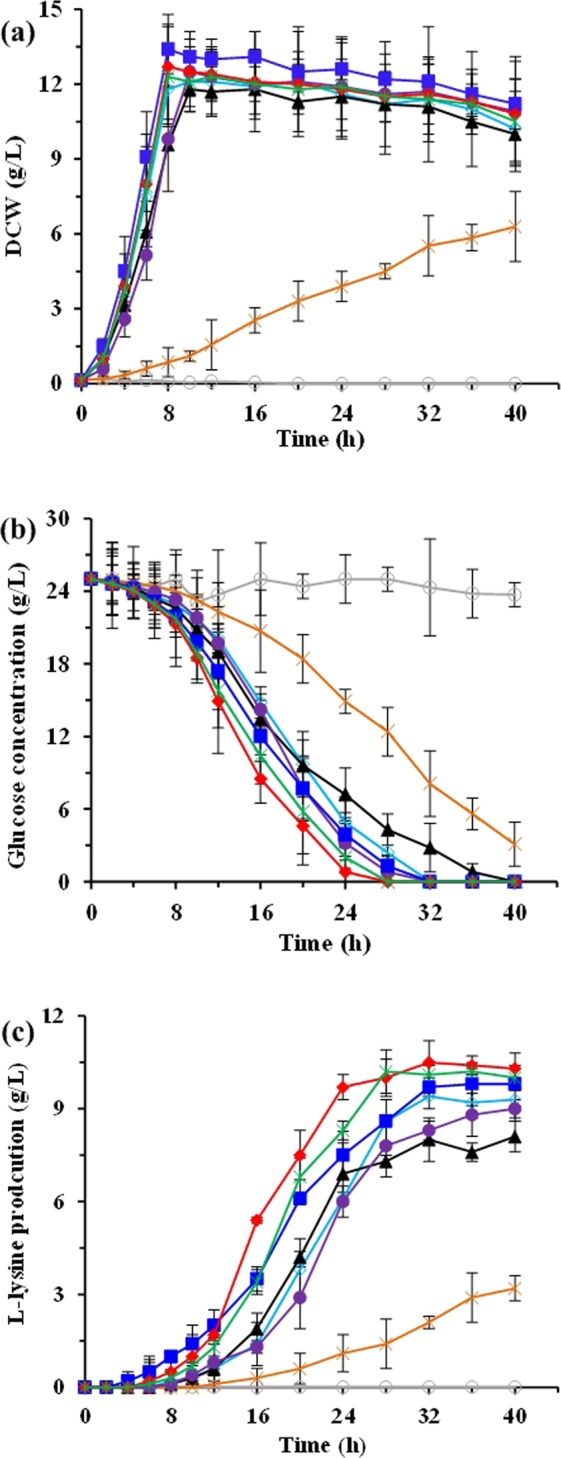
Comparison of cell growth (**a**), glucose (**b**), and L-lysine production (**c**) of different *E. col*i recombinants with different DapDHs in shake-flasks culture with MS medium. Signal denotes: LATR12 (open diamond, sapphire), LATR12-1 (open circle, gray), LATR12-1(*ddh*_Cg_) (triangle, black), LATR12-1(*ddh*_Bs_) (squares, blue), LATR12-1(*ddh*_Ct_) (circle, purple), LATR12-1(*ddh*_St_) (diamond, red), LATR12-1(*ddh*_Ut_) (asterisk, green), and LATR12-1(*ddh*_Bf_) (cross, orange). The data represent mean values and standard deviations obtained from three independent cultivations.

As shown in Fig. 1, the succinylase pathway is used as the only pathway for *meso*-DAP biosynthesis catalyzed by four enzymatic steps in *E. coli*. Previous studies^3,23^ have suggested that introduction of the DapDH in L-lysine producer *E. coli* is beneficial to increase the L-lysine production. As mentioned above, six DapDHs from different bacterial are able to catalyze the biosynthesis of *meso*-DAP in *E. coli ∆dapD/∆dapE* (Fig. S3). However, different DapDHs had different temperature optimum and stability (Fig. 2). In addition, our previous work has indicated that the optimal fermentation temperature is 40 °C for producing L-lysine by *E. coli* LATR12 (Fig. S4). To investigate whether the introduction of DapDHs would improve the L-lysine productivity in LATR12, we compared the effects of these DapDHs on L-lysine production in the DapD-deficient strain LATR12-1. Expectedly, heterogeneous expression of DapDHs was able to complement the cell growth and L-lysine production of the LATR12-1 (Fig. 4a,b). However, heterogeneous expression of *Bf*DapDH or *Cg*DapDH had a certain negative role on glucose consumption, cell growth and L-lysine production, especially for *Bf*DapDH. The low activity of *Bf*DapDH is most likely due to its low expression^9^, whereas the inappropriate temperature may be contributed to the low activity of *Cg*DapDH^32^. This speculation has been demonstrated in the analysis of the crude enzymatic activity (Table S2). Hudson *et al*.^9^ pointed out that the low specific activity is an innate property of *Bf*DapDH, whereas the activity of *Cg*DapDH was decreased with increasing the incubation time at 40 °C (Fig. 2b). Conversely, the other DapDHs exhibited great momentum in improving the fermentative performance of LATR12 (Fig. 4). Overexpression of *Bs*DapDH showed the best performance in the maximum specific growth rate (μ_max_; 0.27 h^−1^), followed by the *St*DapDH (0.25 h^−1^), *Ut*DapDH (0.25 h^−1^) and *Ct*DapDH (0.21 h^−1^). Interestingly, LATR12-1(*ddh*_St_) (10.3 ± 0.3 g/L) showed the highest production of L-lysine, whereas the L-lysine production of LATR12-1(*ddh*_Bs_) (9.8 ± 0.5 g/L) was only slightly higher than that of LATR12 (9.3 ± 0.4 g/L)(Fig. 4b). DapDH catalyzes the biosynthesis of *meso*-DAP, which can be used as processor for the biosynthesis of peptidoglycan and L-lysine (Fig. 1)^13^. However, the excessive increase in cell growth is not good for L-lysine production because more carbon source enter into the biosynthesis of peptidoglycan rather than L-lysine. To do this, we conceived that heterogeneous expression of *St*DapDh in *E. coli* is beneficial to construct an L-lysine producer with good productive performance.

### Optimizing the expression mode of *St*DapDH to enhance the carbon flux in diaminopimelate pathway

**Figure 5:**
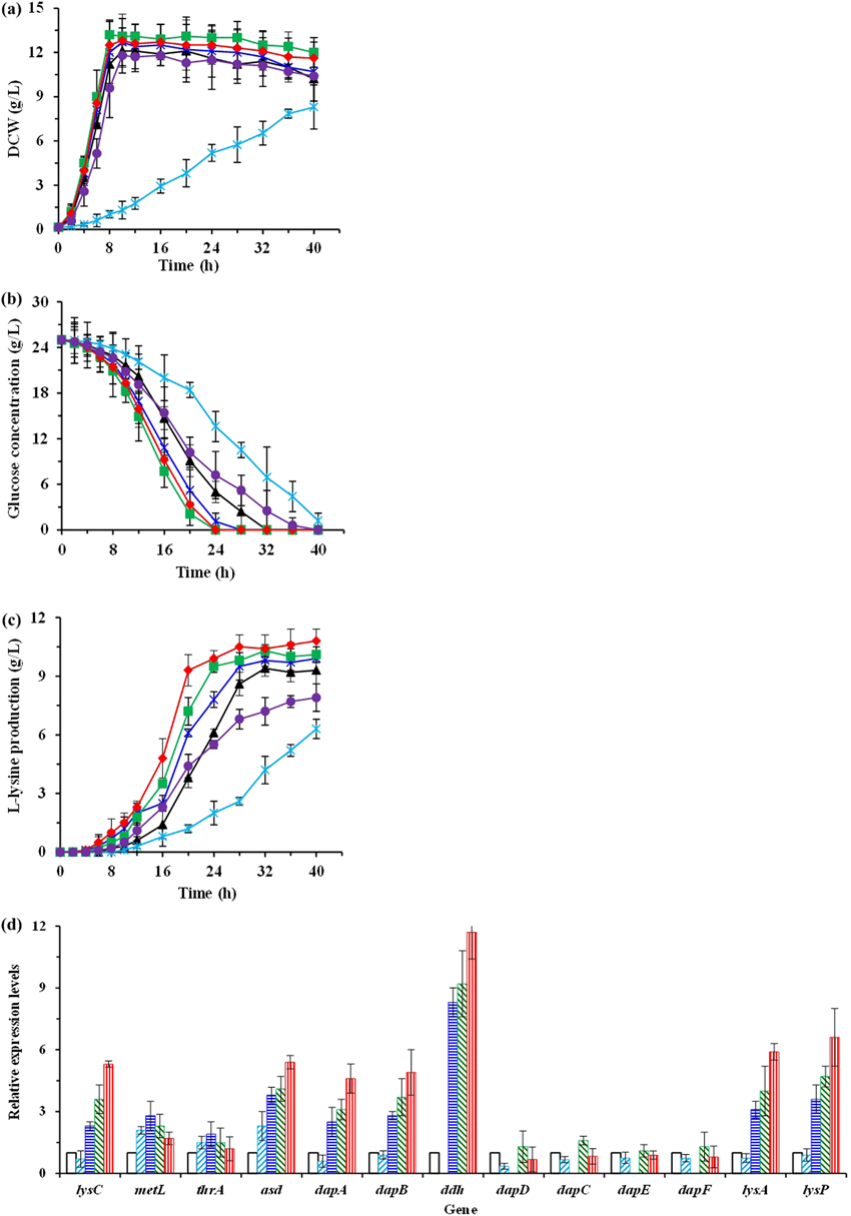
The effects of different integrate modes of *St*DapDH on cell growth (**a**), glucose (**b**), and L-lysine production (**c**) as well as the relative expression levels of genes involved in the L-lysine production (**d**) in shake-flasks culture with MS medium. Signal denotes: LATR12 (∆ or , black), LATR12-2 (* or , sapphire), LATR12∆*dapD::ddh*_St_ (× or , blue), LATR12∆*rpiB::ddh*_St_ (□ or , green), LATR12-2∆*rpiB::ddh*_St_ (◊ or , red), LATR12-2∆*rpiB::ddh*_Cg_ (○, purple). The data represents values and standard deviations obtained from three independent cultivations.

As stated above, *St*DapDH plays a positive role on improving L-lysine production by *E. coli*, but its catalytic efficiency is controlled by nucleotide-cofactor, substrate and product. In this study, we aimed to enhance the L-lysine productivity of LATR12 by optimizing the integrated mode of *St*DapDH-coding gene in LATR12 genome. The integrated modes included three dimensions: (1) the *St*DapDH-coding gene integrates at *dapD* loci of LATR12, resulted a LATR12*∆dapD::ddh*_St_; (2) the *St*DapDH-coding gene integrates at *rpiB* loci of LATR12, resulted a LATR12*∆rpiB::ddh*_St_; (3) the *St*DapDH-coding gene integrates at *rpiB* loci of LATR12-2 with weakened DapD, resulted a LATR12-2*∆rpiB::ddh*_St_. The original strain LATR12 and these recombinant strains were then used to investigate the efficiency of L-lysine fermentation process. Compared with LATR12, the disruption of *rpiB* (encoding ribose-5-phosphate isomerase B, a nonessential enzyme for growth of *E. coli* K12)^33^ did not affect the cell growth and L-lysine production (Fig. S5). The data of glucose consumption and cell growth showed that the integrated mode of *St*DapDH-coding gene did not significantly change the glucose consumption and cell growth (Fig. 5a,b). However, the L-lysine production varied obviously with the change of integrated mode (Fig. 5c). The highest L-lysine production was observed for LATR12-2*∆rpiB::ddh*_St_ (10.8 ± 0.6 g/L), followed by LATR12*∆rpiB::ddh*_St_ (10.1 ± 0.4 g/L) and LATR12*∆dapD::ddh*_St_ (9.9 ± 0.5 g/L). Previous studies^18,24,32^ and our results (Fig. 3f,g) have proved that *meso*-DAP inhibits slightly the amination of THDPA, whereas *L,L*-DAP inhibits the deamination of *meso*-DAP, which are likely to cause more *meso*-DAP into decarboxylation catalyzed by diaminopimelate decarboxylase rather than into deamination because of exist of *L,L*-DAP. In addition, these results reconfirmed that heterogeneous expression of *Cg*DapDh in *E. coli* is not better than that of *St*DapDh for L-lysine production (Fig. 5).

In order to know the reasons of change, we investigated the relative expression level of genes in L-lysine biosynthetic pathway from L-aspartate (i.e., *lysC*, *metL*, *thrA*, *asd*, *dapA*, *dapB*, *ddh*, *dapD*, *dapC*, *dapE*, *dapF*, *lysA* and *lysP*) in original strain and these recombinant strains (Fig. 5d). The levels of transcription of *lysC*, *asd*, *dapA*, *dapB*, *lysA* and *lysP* were significantly increased with introducing *St*DapDH in original strain LATR12. However, the relative expression levels of *metL* and *thrA*, as the bifunctional genes encoding aspartate kinase and homoserine dehydrogenase, were controlled by the expression levels of genes in succinylase pathway. As can be seen from Fig. 5d, *metL* and *thrA* exhibited an increasing expression level only by weakening or deleting *dapD*, whereas their expression levels suddenly decreased in LATR12*∆rpiB::ddh*_St_ and LATR12-2*∆rpiB::ddh*_St_. Expectedly, the expression levels of genes in succinylase pathway (i.e., *dapD*, *dapC*, *dapE* and *dapF*) decreased even disappeared by weakening or deleting *dapD*. Interestingly, the expression levels of genes in succinylase pathway increased slightly with introducing *St*DapDH in original strain LATR12 (Fig. 5d).

### Optimizing the availability of ammonium to improve the production efficiency of L-lysine in recombinant strains

In the course of L-lysine biosynthesis, the ammonium availability is one of greatest importance in either succinylase pathway or dehydrogenase pathway (Fig. 1). However, the ammonium concentration for stimulating the function of dehydrogenase pathway is higher than that of succinylase pathway^9,19^. Assuming that an increased ammonium availability could improve the fermentation performances of LATR12*∆dapD::ddh*_St_, LATR12*∆rpiB::ddh*_St_ and LATR12-2*∆rpiB::ddh*_St_, we optimized the initial concentration of ammonium (i.e., (NH_4_)_2_SO_4_) in MS medium. As shown in Table 2, the maximum L-lysine production, cell growth and α obtained at the initial (NH_4_)_2_SO_4_ concentration of 20 g/L for LATR12-2*∆rpiB::ddh*_St_ (12.3 ± 0.6 g/L of L-lysine) and LATR12*∆rpiB::ddh*_St_ (10.9 ± 0.5 g/L of L-lysine), whereas the optimal (NH_4_)_2_SO_4_ concentration was 25 g/L for LATR12*∆dapD::ddh*_St_ (10.5 ± 0.4 g/L of L-lysine). Although the maximal specific production rate of L-lysine (*q*_Lys, max._) was kept at a higher level at ≥15 g/L of (NH_4_)_2_SO_4_, the L-lysine production, cell growth and α decreased with increasing the (NH_4_)_2_SO_4_ concentration. This is because that the high ammonium concentration inhibits the cell growth (Table 2)^34^. In order to understand the mechanism of ammonium uptake, we investigated the relative expression level of ammonium transporter (AmtB, encoded by *amtB* gene) and its regulatory protein (Uridylyltransferase; UTase, encoded by *glnD* gene) between without (NH_4_)_2_SO_4_ and with 20 g/L of (NH_4_)_2_SO_4_ by semiquantitative RT-PCR (Fig. S6). AmtB (encoded by *amtB*) is the main ammonium uptake system in *E. coli*^35^, but its function is regulated by UTase and a PII-type GlnK protein (for review, see Burkovsk *et al*.)^36^. Consistent with the previous results^37,38^, the expression level of *amtB* was decreasing, whereas the expression level of *glnD* was increasing with the increase of (NH_4_)_2_SO_4_ (Fig. S6). Interestingly, the expression level of *amtB* in cells grown without (NH_4_)_2_SO_4_ was much higher than that of cells grown with 20 g/L of (NH_4_)_2_SO_4_, especially for LATR12*∆dapD::ddh*_St_ (186-fold). Conversely, the expression level of *glnD* in cells grown without (NH_4_)_2_SO_4_ was lower than that of cells grown with 20 g/L of (NH_4_)_2_SO_4_ (Fig. S6). These results showed that LATR12*∆dapD::ddh*_St_ was more sensitive to ammonium concentration than the other test strains.

**Table 2:** The DCW, L-lysine production, carbon yield (α), and maximal specific production rate of L-lysine (*q*_Lys, max._) of genetically defined *E. coli* strains under the different concentration of (NH_4_)_2_SO_4_^a^.

(NH_4_)_2_SO_4_ Conc. (g/L)	LATR12				LATR12*∆dapD::ddh*_St_				LATR12*∆rpiB::ddh*_St_				LATR12-2*∆rpiB::ddh*_St_			
	Lys Conc. (g/L)	DCW (g/L)	α (%)	*q*_Lys, max._ (g/(g∙h))	Lys Conc. (g/L)	DCW (g/L)	α (%)	*q*_Lys, max._ (g/(g∙h))	Lys Conc. (g/L)	DCW (g/L)	α (%)	*q*_Lys, max._ (g/(g∙h))	Lys Conc. (g/L)	DCW (g/L)	α (%)	*q*_Lys, max._ (g/(g∙h))
0	ND	4.7 ± 0.6	—	—	ND	4.5 ± 0.5	—	—	ND	4.6 ± 0.4	—	—	ND	4.5 ± 0.2	—	—
5	1.2 ± 0.1	8.9 ± 0.5	4.8	0.09 ± 0.01	1.2 ± 0.2	9.4 ± 0.4	4.8	0.07 ± 0.01	1.9 ± 0.2	10.7 ± 0.5	7.6	0.11 ± 0.01	2.5 ± 0.4	9.8 ± 0.7	10.0	0.15 ± 0.02
10	4.6 ± 0.5	10.0 ± 0.6	18.4	0.20 ± 0.01	5.2 ± 0.5	10.1 ± 1.0	20.8	0.17 ± 0.03	6.4 ± 0.4	11.5 ± 0.8	25.6	0.22 ± 0.02	7.7 ± 0.7	10.1 ± 0.5	30.8	0.28 ± 0.04
15	8.9 ± 0.4	10.5 ± 0.9	35.6	0.28 ± 0.03	9.3 ± 0.6	10.6 ± 0.4	37.2	0.26 ± 0.05	9.2 ± 0.2	12.1 ± 1.5	36.8	0.31 ± 0.01	10.4 ± 0.3	11.0 ± 0.8	41.6	0.38 ± 0.04
20	8.7 ± 0.7	9.2 ± 0.3	34.8	0.26 ± 0.03	9.8 ± 0.4	12.1 ± 1.3	39.2	0.27 ± 0.03	10.9 ± 0.5	12.4 ± 0.9	43.6	0.36 ± 0.05	12.3 ± 0.6	11.8 ± 1.1	49.2	0.47 ± 0.03
25	7.6 ± 0.2	8.3 ± 0.8	30.4	0.26 ± 0.02	10.5 ± 0.8	12.3 ± 1.8	42.0	0.29 ± 0.02	9.8 ± 1.2	12.0 ± 1.1	39.2	0.35 ± 0.04	11.2 ± 0.8	11.6 ± 10	44.8	0.46 ± 0.04
30	6.0 ± 0.5	6.8 ± 0.2	24.0	0.27 ± 0.04	8.8 ± 0.6	9.8 ± 0.5	35.2	0.30 ± 0.05	8.9 ± 0.4	9.5 ± 0.4	35.6	0.37 ± 0.03	9.5 ± 0.7	9.3 ± 0.9	38.0	0.46 ± 0.03
40	3.3 ± 0.3	4.7 ± 0.6	13.2	0.26 ± 0.03	4.2 ± 0.3	6.5 ± 0.6	16.8	0.29 ± 0.03	4.7 ± 0.2	6.8 ± 0.7	18.8	0.35 ± 0.04	5.2 ± 0.6	6.4 ± 0.4	20.8	0.45 ± 0.03
50	0.5 ± 0.1	2.3 ± 0.2	2.0	0.25 ± 0.02	1.4 ± 0.2	3.6 ± 0.4	5.6	0.32 ± 0.05	1.4 ± 0.1	3.9 ± 0.2	5.6	0.31 ± 0.03	1.7 ± 0.1	3.3 ± 0.2	6.8	0.46 ± 0.05

^a^Lys Conc.: L-lysine concentration; DCW: dry cell weight; ND: Not detected; -: No computed data.

All data are meaning values of three determinations of three independent experiments with ± SD.

### Changes of carbon flux in LATR12, LATR12*∆dapD::ddh*_St_ and LATR12-2*∆rpiB::ddh*_St_

**Figure 6:**
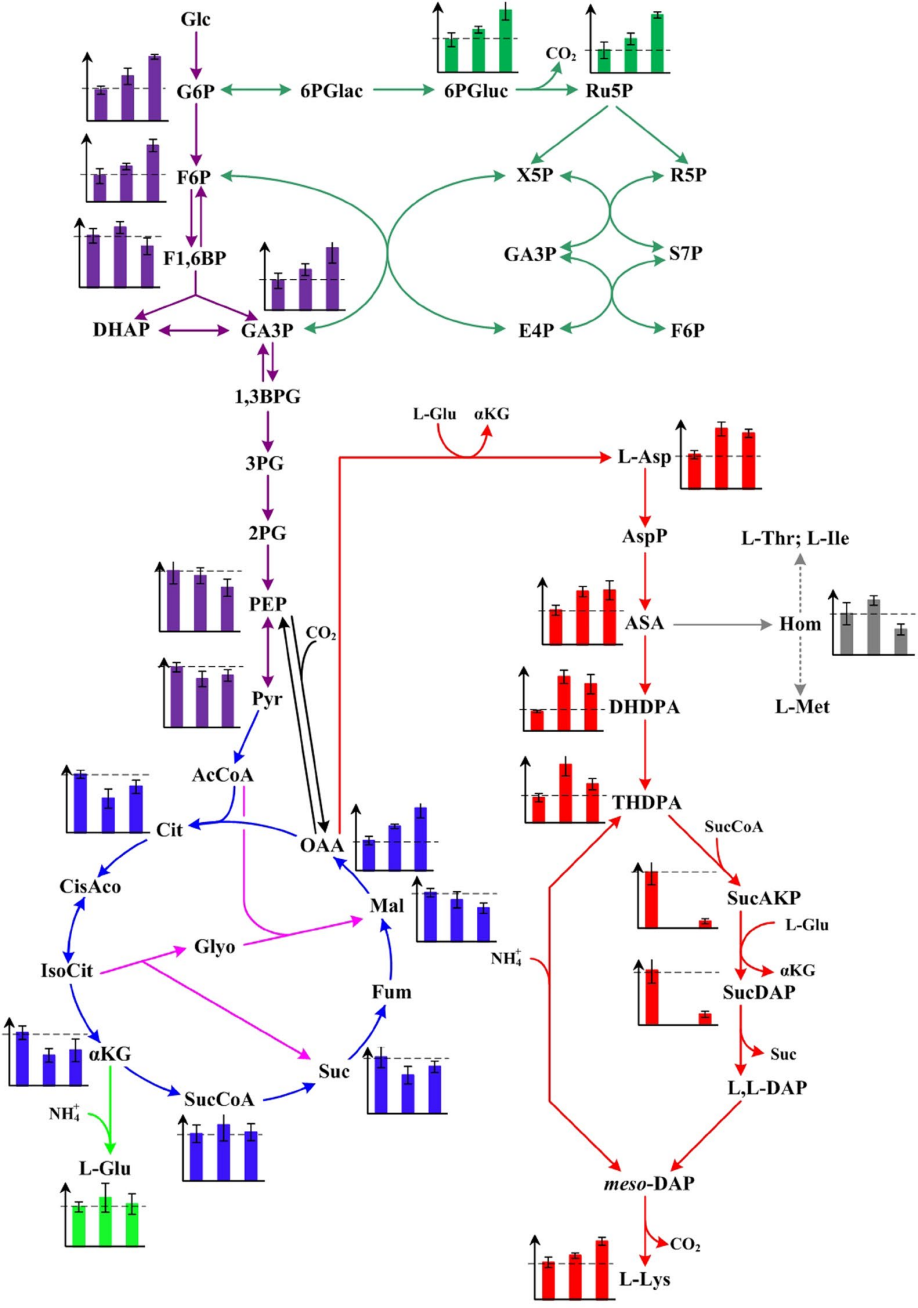
Levels of intermediates involved in L-lysine biosynthesis detected in LATR12, LATR12∆*dapD::ddh*_St_ and LATR12-2∆*rpiB::ddh*_St_. The x-axes represent LATR12, LATR12∆*dapD::ddh*_St_ and LATR12-2∆*rpiB::ddh*_St_. The y-axes represent the relative abundance of intermediate, which was calculated by normalizing the peak area of metabolite against the total peak area within the sample. Abbreviations: *Glc* Glucose, *G6P* Glucose-6-phosphate, *F6P* Fructose-6-phosphate, *F1,6BP* Fructose-1,6-bisphosphate, *DHAP* Dihydroxyacetone phosphate, *GA3P* Glyceraldehydes-3-phosphate, *1,3BPG* 1,3-diphosphoglycerate, *3PG* 3-phosphoglycerate, *2PG* 2-phosphoglycerate, *PEP* Phosphoenolpyruvate, *Pyr* Pyruvate, *AcCoA* Acety-CoA, *Cit* Citrate, *CisAco* Cis-aconitate, *IsoCit*, isocitrate; *α-KG*, α- ketoglutarate, *SucCoA*, Succinyl-CoA, *Suc* Succinate, *Fum* Fumarate; *Mal* Malate, *OAA* Oxaloacetate, *L-Glu* L-glutamate, *6PGlac* 6-phosphoglucono-1,5-lactone, *6PGluc* 6-phosphogluconate, *Ru5P* Ribulose-5-phosphate, *X5P* Xylulose-5-phosphate, *R5P* Ribose-5-phosphate, *S7P* Sedoheptulose-7-phosphate, *E4P* Erythrose-4-phosphate, *L-Asp* L-aspartate, *AspP* L-aspartate phosphate, *ASA* L-aspartate-4-semialdehyde, *DHDPA* L-2,3-dihydrodipicolinate, *THDPA* L-∆^1^-Tetrahydrodipicolinate, *SucAKP* L-*N*-Succinyl-2-amino-6-ketopimelate, *SucDAP N*-succinyl-L,L-2,6-diaminopimelate, *L,L-DAP* L,L-diaminopimelate, *meso-DAP meso*-diaminopimelate, *L-Lys* L-lysine.

As mentioned above, introducing the *St*DapDH in DapD-deficient or DapD-attenuated strain increased significantly the performance of L-lysine production as compared with the original strain LATR12. To study the effects of *St*DapDH on L-lysine production, the changes of carbon flux in LATR12, LATR12*∆dapD::ddh*_St_ and LATR12-2*∆rpiB::ddh*_St_ were studied using GC-MS. More than 70 intracellular metabolites showed different levels in LATR12, LATR12*∆dapD::ddh*_St_ and LATR12-2*∆rpiB::ddh*_St_. Among these 70 metabolites, 23 of them were closely related to L-lysine production in the biosynthetic pathway. To get a more detailed view of the changes in carbon flux caused by the introduction of *St*DapDH, the relative content of these 23 metabolites were determined in the post-logarithmic phase (Table S3). As shown in Fig. 6, the content of intermediates in pentose phosphate (PP) pathway including glucose-6-phosphate, frucose-6-phosphate and glyceraldehydes-3-phosphate were higher, but the content of phosphoenolpyruvate and pyruvate as the substrates of carbon anaplerotic reaction were slightly lower in recombination strains than in LATR12. It has been proven that 4 mol of NADPH is required for the production of 1 mol of L-lysine, and PP pathway is generally considered major pathway for NADPH formation^1^. This is why introduction of *St*DapDH led to elevated levels of PP pathway intermediates. The decrease of phosphoenolpyruvate and pyruvate could potentially be linked to the original strain used in the study (Table 3). MF disrupts the TCA cycle, and the MF-resistant mutants show a higher activity of phosphoenolpyruvate carboxylase^39^. However, the content of intermediates in TCA cycle was decreased during introduction of *St*DapDH in LATR12 except succinyl-CoA and oxaloacetate (OAA), which are the co-precursors for L-lysine biosynthesis (Fig. 6). Previous results indicated that the L-lysine biosynthetic pathway becomes even more efficient because of introduction of *St*DapDH^3,23^. From which we can infer that more carbon flux flow into OAA. In addition, Kind *et al*.^40^ pointed out that succinyl-CoA serves as precursor for L-lysine biosynthesis via succinylase pathway. This may be the reason for the increase of succinyl-CoA in LATR12*∆dapD::ddh*_St_ and LATR12-2*∆rpiB::ddh*_St_, in which dehydrogenase pathway is the main pathway for L-lysine biosynthesis. As another co-precursor, the content of L-glutamate was slightly higher in recombination strains than in LATR12. Predictably, the content of intermediates in terminal pathway of L-lysine biosynthesis was dramatically increasing in recombination strains except L-*N*-Succinyl-2-amino-6-ketopimelate and *N*-Succinyl-L,L-2,6-diaminopimelate, which are the intermediates in succinylase pathway. It should be noted that L-homoserine, as by-product of L-lysine production, in LATR12*∆dapD::ddh*_St_ was higher than that in LATR1 and LATR12-2*∆rpiB::ddh*_St_ (Fig. 6).

**Table 3:** Strains used in this study.

*E. coli* strains	Relevant characteristic(s)	Reference
BL21 (DE3)	F^−^ *ompT gal dcm lon hsdS*_*B*_ (r_B_ m_B_) λ(DE3)	Stratagene
LATR11	L-lysine producer *E. coli* AEC^hr^ Thr^−^ Rif^r^, derived from *E. coli* MG1655	^3^
LATR12	L-lysine producer *E. coli* AEC^hr^ Thr ^–^ Rif^r^ MF^r^, derived from *E. coli* LATR11	Our Lab
*∆dapD/∆dapE*	Knockout the natural *dapD* and *dapE* gene in strain *E. coli* MG1655 chromosome	Our Lab
BL21 pET28a/*ddh*_Cg_	*E. coli BL21 harboring the plasmid* pET28a/*ddh*_Cg_	This work
BL21 pET28a/*ddh*_Bf_	*E. coli BL21 harboring the plasmid* pET28a/*ddh*_Bf_	This work
BL21 pET28a/*ddh*_Ct_	*E. coli BL21 harboring the plasmid* pET28a/*ddh*_Ct_	This work
BL21 pET28a/*ddh*_Bs_	*E. coli BL21 harboring the plasmid* pET28a/*ddh*_Bs_	This work
BL21 pET28a/*ddh*_St_	*E. coli BL21 harboring the plasmid* pET28a/*ddh*_St_	This work
BL21 pET28a/*ddh*_Ut_	*E. coli BL21 harboring the plasmid* pET28a/*ddh*_Ut_	This work
LATR12*∆dapD* (or LATR12-1)	Knockout the natural *dapD* gene in strain LATR12 chromosome	This work
LATR12-1(*ddh*_Cg_)	*E. coli* LATR12 *∆dapD harboring the plasmid* pDXW-8/*ddh*_Cg_	This work
LATR12-1(*ddh*_Bf_)	*E. coli* LATR12 *∆dapD harboring the plasmid* pDXW-8/*ddh*_Bf_	This work
LATR12-1(*ddh*_Ct_)	*E. coli* LATR12 *∆dapD harboring the plasmid* pDXW-8/*ddh*_Ct_	This work
LATR12-1(*ddh*_Bs_)	*E. coli* LATR12 *∆dapD harboring the plasmid* pDXW-8/*ddh*_Bs_	This work
LATR12-1(*ddh*_St_)	*E. coli* LATR12 *∆dapD harboring the plasmid* pDXW-8/*ddh*_St_	This work
LATR12-1(*ddh*_Ut_)	*E. coli* LATR12 *∆dapD harboring the plasmid* pDXW-8/*ddh*_Ut_	This work
LATR12*dapD*^A1G^ (or LATR12-2)	Replacement of the start code ATG by GTG in the *dapD* gene of strain LATR12 chromosome	This work
LATR12*∆dapD::ddh*_St_	Replacement of the natural *dapD* gene with the *ddh*_St_ cassette in strain LATR12 chromosome	This work
LATR12*∆rpiB::ddh*_St_	Replacement of the natural *rpiB* gene with the *ddh*_St_ cassette in strain LATR12 chromosome	This work
LATR12-2*∆rpiB::ddh*_St_	Replacement of the natural *rpiB* gene with the *ddh*_St_ cassette in strain LATR12-2 chromosome	This work
LATR12-2*∆rpiB::ddh*_Cg_	Replacement of the natural *rpiB* gene with the *ddh*_Cg_ cassette in strain LATR12-2 chromosome	This work

In conclusion, the intermediates in NADPH biosynthetic pathway (e.g., gluconolactone-6-phosphate and ribulose-5-phosphate), the precursors of L-lysine (e.g., L-glutamate and OAA) and the intermediates in terminal pathway of L-lysine biosynthesis (e.g., L-aspartate-4-semialdehyde and L-∆^1^-Tetrahydrodipicolinate) were increased, whereas the intermediates in by-products biosynthetic pathway (e.g., succinate and homoserine) were decreased in LATR12*∆dapD::ddh*_St_ and LATR12-2*∆rpiB::ddh*_St_, because more carbon source should be used for L-lysine production during introduction of *St*DapDH.

### Fed-batch fermentation of LATR12 and LATR12-2*∆rpiB::ddh*_St_

**Figure 7:**
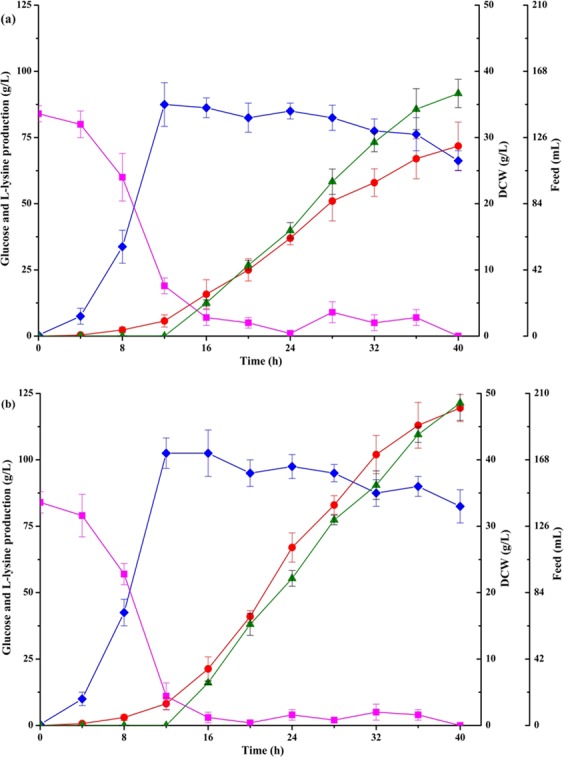
Time course of L-lysine fed-batch fermentations of strains LATR12 (**a**) and LATR12-2∆*rpiB::ddh*_St_ (**b**) in 5-L fermentors. Fed-batch cultivation was performed with an initial glucose concentration of 80 g L^−1^. The residual glucose concentration in the fermentation broth was maintained constantly (5~10 g/L) by monitoring the residual glucose concentration of the broth and controlling the feed rate. Signal denotes: DCW (◆, blue), Glucose (■, pink), L-lysine (●, red) and feed (▲, green). The data represent mean values and standard deviations obtained from three independent cultivations.

The production performance of strains LATR12 and LATR12-2*∆rpiB::ddh*_St_ was investigated in a fed-batch process. Figure 7 shows the time profiles of fed-batch fermentations in a 5-L jar fermenter. Fed-batch fermentation of LATR12-2*∆rpiB::ddh*_St_ resulted in 119.5 ± 7.2 g/L of L-lysine with a productivity of 2.99 g/(L∙h) and carbon yield of 49.1%. However, fed-batch fermentation of LATR12 resulted in 71.8 ± 5.2 g/L of L-lysine with a productivity of 1.80 g/(L∙h) and carbon yield of 35.3%. Consistent with the results of GC-MS in shake flasks, the yield of phosphoenolpyruvate, pyruvate, α-ketoglutarate and succinate were lower, but the yield of succinyl-CoA and oxaloacetate were higher in LATR12-2*∆rpiB::ddh*_St_ than in LATR12 (Table S4). Moreover, the yield of L-methionine was lower, whereas the yield of L-glutamate was slightly higher in LATR12-2*∆rpiB::ddh*_St_ than in LATR12 (Table S4). Thus, the final strain LATR12-2*∆rpiB::ddh*_St_ also allowed efficient L-lysine production under fed-batch fermentation.

## Conclusions

For the first time, introduction of DapDH with high temperature optimum was identified as a critical factor for efficiently producing L-lysine in *E. coli*. It is clear from the study of the functions and kinetic properties of DapDHs that different DapDHs show a huge difference in the oxidative deamination and reductive amination, and show a higher catalytic efficiency for reductive amination than for oxidative deamination except the *Bf*DapDH (Tables 1 and S1). In addition, different DapDHs show different responses for nucleotide-cofactor, substrate and product. For example, the activity of *Ct*DapDH and *Bs*DapDH was dramatically decreasing with the increase of the concentration of NADP^+^, whereas the *Cg*DapDH showed the minimal changes. In addition, the integrated mode and ammonium availability were also investigated, indicating that the co-existence of two pathways and sufficient ammonium availability are good for increasing the final titer of L-lysine with a high carbon yield and productivity in *E. coli*. Fed-batch fermentation of the target strain LATR12-2*∆rpiB::ddh*_St_ resulted in 119.5 ± 7.2 g/L of L-lysine with a carbon yield of 49.1% and productivity of 2.99 g/(L∙h). These results indicated that overexpression of thermostable *St*DapDH to redirect diaminopimelate pathway has great potential to improve the efficiency of L-lysine production in *E. coli*. Although the efficiency of L-lysine production of LATR12-2*∆rpiB::ddh*_St_ is relatively low so that it should not be used for the practical industrial application level, the L-lysine yield and productivity are higher than those reported in literature (Table S5)^21,34,41,42^. Thus, the final strain LATR12-2*∆rpiB::ddh*_St_ has great potential for industrial L-lysine production. Because the genetic modification was integrated into the genome such that the strain is stable and production does not need the selection markers except for the relatively high L-lysine production. In order to further increase the efficiency of L-lysine production of LATR12-2*∆rpiB::ddh*_St_, the carbon flux partitioning in metabolic network need improvement in the next work, for example, forcing more flux into L-lysine pathway and minimizing the carbon loss. In addition, to improve and optimize NADPH availability is also one of the most effective ways to improve L-lysine production, for which multiple strategies are available (for review, see Xu *et al*.)^1^.

## Methods

### Strains, growth medium and culture conditions

Strains used in this study are listed in Table 3. L-lysine producing strain *E. coli* LATR12 (i.e., *E. coli* AEC^hr^ Thr ^–^ Rif ^r^ MF ^r^) was derived from the wild-type strain *E. coli* MG1655, which was mutagenized by atmospheric and room temperature plasma (ARTP) biological breeding system (Si Qing Yuan Biotechnology Co., Ltd, Beijing, China). *E. coli* LATR12 was resistant to rifampicin (Rif ^r^)0, monfluoroacetate (MF ^r^) and s-2-aminoethyl- L-cysteine (AEC^hr^), and was L-threonine auxotroph (Thr^−^).

The growth medium and culture conditions were illustrated in “Supplementary Info File”.

### Protein expression, purification and activity assay

The recombinant *E. coli* cell were grown overnight at 37 °C with shaking at 120 r/min in 10 mL of LB with 50 µg/mL of Km. For overexpression, the procedure was performed according to the description of Xu *et al*.^5^. The cultures were centrifuged to obtain the cell pellets at 5000 × *g*, and were lysed by sonication (Sonics & Materials, Inc., Connecticut, USA). Subsequently, the mixture was purified as previously described by Trigoso *et al*.^43^. SDS-PAGE was used to analyze the purity of DapDH after purified by affinity chromatography. The enzyme activity assay is stated in “Supplementary Info File”.

### Construction of *E.coli* recombinant strains

The gene deletions and gene replacements were executed in *E. coli* chromosome was performed by the published method^44^. The procedures of recombinant strain construction were illustrated in “Supplementary Info File”. Plasmids and oligonucleotides used in this study are listed in Tables S6 and S7, respectively. The target recombinant strains were selected according to the procedures described by Link *et al*.^44^. The deletions in the chromosome were verified by PCR analysis with the corresponding primer pairs, respectively (Table S7). The gene replacements were validated via sequencing by Sangon Biotech (Shanghai) Co., Ltd. (Shanghai, China). The detail of DNA manipulations and transformations are stated in “Supplementary Info File”.

### RNA isolation and quantitative real-time PCR (qRT-PCR)

Total cellular RNA was extracted from cells at the exponential phase using the total RNA extraction kit as described by the manufacturer (BioFlux, Beijing, China). RNA preparations were treated with DNase I to eliminate residual DNA. The cDNA was synthesized using RevertAid^TM^ First Strand cDNA synthesis kit (Fermentas, Shanghai, China). The qRT-PCR was performed using the QIAGEN OneStep RT-PCR Kit (TIANGEN, Beijing, China) on iCycler iQ5 real-time PCR system (Bio-Rad, Richmond, USA). The PCR reaction system and procedure was set following our previous reports^5^. The transcriptional levels were normalized to the 16S rRNA from the same RNA samples. Each sample was analyzed in triplicate.

### Analytical methods

A sample was taken from the shake flasks or fermenter every 2 or 4 h. A half of sample was used to measure the biomass concentration using a spectrophotometer at 600 nm or by gravimetric analysis. The correlation factor between dry cell weight (DCW) and OD_600_ was determined as 0.277 (1 OD_600_ = 0.277 g DCW). The other half of sample was diluted 100-fold, and then used to determine the glucose and l-lysine concentration using an SBA-40E immobilized enzyme biosensor (Shandong, China). The intracellular metabolites of different strains were analyzed by gas chromatography-mass spectrometry (GC-MS) according to the previous described^45^. By the end of fermentation, the fermentation liquors were also used to determine the concentration of by-products (including amino acids and organic acids) by high performance liquid chromatography (HPLC) according to the procedure described by Xu, *et al*.^46^. All data were collected from three independent culture samples, and then were analyzed statistically by Student’s *t* test with a two-tailed distribution.

## Supplementary information

Supplementary Info File
