# Supplementary material for: Overexpression of thermostable meso-diaminopimelate dehydrogenase to redirect diaminopimelate pathway for increasing L-lysine production in Escherichia coli
Source: Sci Rep. 2019 Feb 20;9:2423. doi: 10.1038/s41598-018-37974-w (PMC6382763; doi:10.1038/s41598-018-37974-w)
Supplement: Supplementary file 1 — Supplementary Info File [file 41598_2018_37974_MOESM1_ESM.docx]

**Overexpression of thermostable *meso*-diaminopimelate dehydrogenase to redirect diaminopimelate pathway for increasing L-lysine production in *Escherichia coli***

**Running Title:** Thermostable DapDH benefits increasing L-lysine biosynthesis

**Jian-Zhong Xu ^1, *^, Hao-Zhe Ruan ^1^, Li-Ming Liu ^2^, Lu-Ping Wang ^1^, Wei-Guo Zhang ^1^**

^1^ The Key Laboratory of Industrial Biotechnology, Ministry of Education, School of Biotechnology, Jiangnan University, 1800^#^ Lihu Road, WuXi 214122, People’s Republic of China

^2^ State Key Laboratory of Food Science and Technology, School of Biotechnology, Jiangnan University, 1800^#^ Lihu Road, WuXi 214122, People’s Republic of China

^*^ Corresponding authors:

Jian-Zhong Xu; E-mail: [xujianzhong@jiangnan.edu.cn](mailto:xujianzhong@jiangnan.edu.cn); Tel: +86-510-85329312; Fax: +86-510-85329312

**Growth medium and culture conditions**

The growth media, including Luria-Bertani (LB), SOB, SOC, MS, seed medium and fermentation medium, were prepared according to the description of [Imaizumi, et al. ^1^](#_ENREF_1) and [Xu, et al. ^2^](#_ENREF_2). The analysis of the effect of ammonium availability was carried out by only varying the (NH_4_)_2_SO_4_ concentration in the MS medium. The selective media, including chloramphenicol-LB and 5% (wt/vol) sucrose+kanamycin-LB, were prepared according to the description of [Link, et al. ^3^](#_ENREF_3). Appropriately, 0.2 g/L l-threonine was added in the MS and seed medium, whereas 0.4 g/L l-threonine was added in the fermentation medium. In addition, 50 μg/mL of kanamycin (Km) or 25 μg/mL of chloramphenicol (Cm) was used to obtain recombinant strains, and 1 mmol/L of isopropyl β-D-1-thiogalactopyranoside (IPTG) was used to induce gene overexpression. Batch cultivation in shake flasks and fed-batch fermentation in 5-L jar fermenter (BLBio-5GJ-2-H, Bailun Bi-Technology Co. Ltd., Shanghai, China) was carried out as described by [Xu, et al.](#_ENREF_2) ^[2](#_ENREF_2)^. Glucose solution, containing sterile 800 g/L of glucose and 400 g/L of (NH_4_)_2_SO_4_, was used to maintain the residual glucose concentration at 5~10 g/L by adjusting the feed rate.

**Enzyme activity assay**

The crude enzyme was prepared according to the procedures reported by [Trigoso, et al.](#_ENREF_4) ^[4](#_ENREF_4)^. After centrifugation at 4°C for 30 min at 10000×*g*, the cell-free supernatants were immediately used to determine the enzyme activities. Protein concentrations were determined using the Bradford Protein Quantification Kit (Sangon, Shanghai, China) with bovine serum albumin as standard. The activities of DapDH was detected according to the protocol of [Hudson, et al. ^5^](#_ENREF_5) with some modification: 200mmol/L glycine-KOH (pH 10.5) was replaced by Tris-HCl (pH 7.5). Tetrahydrodipicolinate N-succinyltransferase (DapD) was detected according to the protocol of [Schnell, et al.](#_ENREF_6) ^[6](#_ENREF_6)^. Kinetic analysis of DapDH was carried out by varying the substrate concentration (including THDPA, $\mathrm{NH}_{4}^{+}$ and *meso*-DAP) with NADPH or NADP^+^ as cofactor. The analyses were done in triplicate, and all analyses were performed at 30°C unless noted otherwise.

**DNA manipulations and transformations**

Plasmids and oligonucleotides used in this study are listed in Table S6 and Table S7, respectively. The *ddh* gene from different bacteria was chemo-synthesized by General Biosystems (Anhui), Inc. (Chuzhou, China), and then ligated into pMD19-T simple vector to designate as pMD19-T/*ddh*_X_ (*ddh*_X_ indicates the *ddh* gene from different bacteria; Table S6). The target *ddh*_X_ gene segments were amplified using Phusion high-fidelity DNA polymerase (Finnzyme, Espoo, Finland) from pMD19-T/*ddh*_X_. The plasmid construction was illustrated in Supplementary material. The plasmid transformation was performed according to the descriptions of previous reports ^[2](#_ENREF_2)^. Plasmids were extracted from *E. coli* using the SanPrep Mini Plasmid Kit (Sangon, Shanghai, China) and confirmed by restriction endonuclease reaction.

**The procedures of integration vectors constructions**

**The expression vectors pET-28a/*ddh*_Cg_, pET-28a/*ddh*_Bf_, pET-28a/*ddh*_Ct_, pET-28a/*ddh*_Bs_, pET-28a/*ddh*_St_ and pET-28a/*ddh*_Ut_ construction**

The gene *ddh*_X_ from pMD19-T/*ddh*_X_ was amplified by PCR with the corresponding primer pairs, respectively (Table S2). The resulting fragments were purified by the SanPrep DNA Gel Extraction Kit. The gene *ddh*_X_ was purified and digested by suitable restriction enzyme (Table S2), respectively, and then were ligated into pET-28a (+) which was similarly digested. The resulting plasmid was designated as pET-28a/*ddh*_Cg_, pET-28a/*ddh*_Bf_, pET-28a/*ddh*_Ct_, pET-28a/*ddh*_Bs_, pET-28a/*ddh*_St_ and pET-28a/*ddh*_Ut_, respectively*.*

**The expression vectors pDXW-8/*ddh*_Cg_, pDXW-8/*ddh*_Bf_, pDXW-8/*ddh*_Ct_, pDXW-8/*ddh*_Bs_, pDXW-8/*ddh*_St_ and pDXW-8/*ddh*_Ut_ construction**

The gene *ddh*_X_ from pMD19-T/*ddh*_X_ was amplified by PCR with the corresponding primer pairs, respectively (Table S2). The resulting fragments were purified by the SanPrep DNA Gel Extraction Kit. The gene *ddh*_X_ was purified and digested by suitable restriction enzyme (Table S2), respectively, and then were ligated into pDXW-8 which was similarly digested. The resulting plasmid was designated as pDXW-8/*ddh*_Cg_, pDXW-8/*ddh*_Bf_, pDXW-8/*ddh*_Ct_, pDXW-8/*ddh*_Bs_, pDXW-8/*ddh*_St_ and pDXW-8/*ddh*_Ut_, respectively*.*

**The integration vectors pKO3-Km/*dapD*^A1G^ construction**

The *dapDS* fragment (part of *dapD* operon and the upper area of *dapD* operon) from *E. coli* were amplified by PCR with primer pair *dapDS*-F/*dapDS*-R. The resulting fragments were purified by the SanPrep DNA Gel Extraction Kit, and ligated into pMD^TM^ 18T vector via T-A cloning resulting in pMD^TM^ 18T/*dapDS*. The A→G mutation of fragment *dapDS* at loci 1 was carried out by overlap extension PCR with plasmid pMD^TM^ 18T/*dapDS* as template and *dapD*_M_-F/*dapD*_M_-R as primer. The mutation positive plasmid was extracted from positive strain, and digested with *Sal*I/*Not*I, and then were orderly ligated into pKO3-Km which was similarly digested. The resulting plasmid was designated as pKO3-Km/*dapD*^A1G^*.*

**The integration vectors pKO3-Km/∆*dapD* and pKO3-Km/∆*dapD::ddh*_St_ construction**

The *dapD* left (*dapD*-L) and right (*dapD*-R) arms from *E. coli* were amplified by PCR with the corresponding primer pairs, respectively (Table S2). The resulting fragments were purified by the SanPrep DNA Gel Extraction Kit. The fragments of *dapD*-L/ *dapD*-R were purified and digested by suitable restriction enzyme (Table S2), respectively, and then were orderly ligated into pKO3-Km which was similarly digested. The resulting plasmid was designated as pKO3-Km/*∆dapD.*

For construction of pKO3-Km/∆*dapD::ddh*_St_, the cassette of *ddh*_St_ gene was amplified with high-fidelity *pyrobest* DNA polymerase (TAKARA, Dalian, China) from the plasmids pDXW-8/*ddh*_St_ by *P*_tac_-F/*P*_tac_-R. The fragment was purified and digested by suitable restriction enzyme, and then was ligated into pKO3-Km/*∆dapD* which was similarly digested (Table S2), then the resulting plasmid was designated as pKO3-Km/∆*dapD::ddh*_St_*.*

**The integration vectors pKO3-Km/∆*rpiB*, pKO3-Km/∆*rpiB::ddh*_Cg_ and pKO3-Km/∆*rpiB::ddh*_St_ construction**

The *rpiB* left (*rpiB*-L) and right (*rpiB*-R) arms from *E. coli* were amplified by PCR with the corresponding primer pairs, respectively (Table S2). The resulting fragments were purified by the SanPrep DNA Gel Extraction Kit. The fragments of *rpiB*-L/*rpiB*-R were purified and digested by suitable restriction enzyme (Table S2), respectively, and then were orderly ligated into pKO3-Km which was similarly digested. The resulting plasmid was designated as pKO3-Km/∆*rpiB.*

For construction of pKO3-Km/∆*rpiB::ddh*_Cg_ or pKO3-Km/∆*dapD::ddh*_St_, the cassette of *ddh*_Cg_ or *ddh*_St_ gene was amplified with high-fidelity *pyrobest* DNA polymerase (TAKARA, Dalian, China) from the plasmids pDXW-8/*ddh*_Cg_ or pDXW-8/*ddh*_St_ by *P*_tac_-F/*P*_tac_-R, respectively. The fragment was purified and digested by suitable restriction enzyme, and then was ligated into pKO3-Km/∆*rpiB* which was similarly digested (Table S2), then the resulting plasmids were designated as pKO3-Km/∆*rpiB::ddh*_Cg_ and pKO3-Km/∆*rpiB::ddh*_St_*.*

**The procedures of recombinant strain construction**

**Construction of recombinant *E. coli* harboring a series of recombinant pET28a (+) with target gene**

The expression plasmids pET-28a/*ddh*_Cg_, pET-28a/*ddh*_Bf_, pET-28a/*ddh*_Ct_, pET-28a/*ddh*_Bs_, pET-28a/*ddh*_St_ and pET-28a/*ddh*_Ut_ were transformed into competent *E. coli* BL21 (DE3) cells, respectively, then the resulting recombinant strain was designated as *E. coli* pET-28a/*ddh*_Cg_, *E. coli* pET-28a/*ddh*_Bf_, *E. coli* pET-28a/*ddh*_Ct_, *E. coli* pET-28a/*ddh*_Bs_, *E. coli* pET-28a/*ddh*_St_ and *E. coli* pET-28a/*ddh*_Ut_, respectively.

**Construction of *E. coli* LATR12*∆dapD* (i.e., *E. coli* LATR12-1)**

The integration vector pKO3-Km/∆*dapD* was electro-transformed into competent *E. coli* LATR12 cells, and then the resulting recombinant strain was designated *E. coli* LATR12*∆dapD* (i.e., *E. coli* LATR12-1).

**Construction of *E. coli* LATR12*dapD*^A1G^ (i.e., *E. coli* LATR12-2)**

The integration vector pKO3-Km/*dapD*^A1G^ was electro-transformed into competent *E. coli* LATR12 cells, and then the resulting recombinant strain was designated *E. coli* LATR12*dapD*^A1G^ (i.e., *E. coli* LATR12-2).

**Construction of recombinant *E. coli* LATR12∆*dapD* harboring a series of recombinant pDXW-8 with target gene**

The expression plasmids pDXW-8/*ddh*_Cg_, pDXW-8/*ddh*_Bf_, pDXW-8/*ddh*_Ct_, pDXW-8/*ddh*_Bs_, pDXW-8/*ddh*_St_ and pDXW-8/*ddh*_Ut_ were transformed into competent *E. coli* LATR12-1 cells, respectively, and then the resulting recombinant strain was designated as *E. coli* LATR12-1(*ddh*_Cg_), *E. coli* LATR12-1(*ddh*_Bf_), *E. coli* LATR12-1(*ddh*_Ct_), *E. coli* LATR12 LATR12-1(*ddh*_Bs_), *E. coli* LATR12-1(*ddh*_St_) and *E. coli* LATR12-1(*ddh*_Ut_), respectively.

**Construction of *E. coli* LATR12*∆dapD::ddh*_St_, *E. coli* LATR12*∆rpiB::ddh*_St_, *E. coli* LATR12-2*∆rpiB::ddh*_Cg_ and *E. coli* LATR12-2*∆rpiB::ddh*_St_**

The integration vectors pKO3-Km/∆*dapD::ddh*_St_ and pKO3-Km/∆*rpiB::ddh*_St_ were transformed into competent *E. coli* LATR12 cells, respectively, and then the resulting recombinant strain was designated as *E. coli* LATR12∆*dapD::ddh*_St_, *E. coli* LATR12∆*rpiB::ddh*_Cg_ and *E. coli* LATR12∆*rpiB::ddh*_St_, respectively. In addition, the integration vectors pKO3-Km/∆*rpiB::ddh*_Cg_ and pKO3-Km/∆*rpiB::ddh*_St_ were transformed into competent *E. coli* LATR12-2 cells, and then the resulting recombinant strain was designated as *E. coli* LATR12-2*∆rpiB::ddh*_Cg_ and *E. coli* LATR12-2∆*rpiB::ddh*_St_.

**Table S1** The DapDH activity of purified DapDH from different bacteria via the standard assay at 30℃. The oxidization of NADPH or reduction of NADP^+^ was followed and recorded at 340 nm. The data represent mean values and standard deviations obtained from at least three independent measurements with different batches of purified enzymes.

| Enzymes | Specific activity (U/mg protein) | |
| --- | --- | --- |
|  | NADP^+^ | NADPH |
| *Cg*DapDH | 2.96 ± 0.23 | 4.61 ± 0.34 |
| *Bs*DapDH | 2.79 ± 0.28 | 5.72 ± 0.61 |
| *Ct*DapDH | 0.34 ± 0.05 | 0.55 ± 0.03 |
| *St*DapDH | 1.78 ± 0.24 | 2.15 ± 0.12 |
| *Bf*DapDH | 0.51 ± 0.07 | 0.32 ± 0.01 |
| *Ut*DapDH | 1.45 ± 0.11 | 2.40 ± 0.25 |

**Table S2** The DapDH activity of crude DapDH from different bacteria at different point of culture time via the standard assay at 40℃. The oxidization of NADPH was followed and recorded at 340 nm. The data represent mean values and standard deviations obtained from at least three independent measurements with different batches of purified enzymes.

| Strain | Enzyme | Specific activity (U/mg protein) | | |
| --- | --- | --- | --- | --- |
|  |  | 8 h | 16 h | 32 h |
| LATR12-1(*ddh*_Cg_) | *Cg*DapDH | 0.56 ± 0.04 | 0.45 ± 0.03 | 0.30 ± 0.04 |
| LATR12-1(*ddh*_Bs_) | *Bs*DapDH | 0.71 ± 0.02 | 0.66 ± 0.06 | 0.52 ± 0.03 |
| LATR12-1(*ddh*_Ct_) | *Ct*DapDH | 0.15 ± 0.02 | 0.21 ± 0.01 | 0.18 ± 0.02 |
| LATR12-1(*ddh*_St_) | *St*DapDH | 0.55 ± 0.06 | 0.76 ± 0.05 | 0.65 ± 0.04 |
| LATR12-1(*ddh*_Bf_) | *Bf*DapDH | 0.08 ± 0.01 | 0.04 ± 0.02 | 0.02 ± 0.01 |
| LATR12-1(*ddh*_Ut_) | *Ut*DapDH | 0.49 ± 0.05 | 0.65 ± 0.01 | 0.54 ± 0.05 |

**Table S3** The levels of intracellular metabolites involved in L-lysine biosynthesis in LATR12 (Sample A), LATR12*∆dapD::ddh*_St_ (Sample B) and LATR12-2*∆rpiB::ddh*_St_ (Sample C). To ensure the accuracy of the data, five biomass samples of each strain were subjected to GC-MS. Most of the samples were within 95% confidence interval, except sample B1 (dates not show). B1 was judged to be abnormal samples and were omitted from the analysis to ensure the reliability of the results.

| L-Lys | SucDAP | SucAKP | THDPA | DHDPA | Hom | ASA | L-Asp | L-Glu | OAA | Mal | Suc | SucCoA | αKG | Cit | Ru5P | 6PGluc | Pyr | PEP | GA3P | F1,6BP | F6P | G6P | Metabolite |
| --- | --- | --- | --- | --- | --- | --- | --- | --- | --- | --- | --- | --- | --- | --- | --- | --- | --- | --- | --- | --- | --- | --- | --- |
| 7.078192 | 0.203637 | 0.113178 | 0.041847 | 0.085632 | 0.007925 | 0.035732 | 0.021458 | 1.553241 | 0.027638 | 0.050132 | 3.330789 | 0.090684 | 0.023708 | 0.042374 | 0.009564 | 0.016235 | 0.603512 | 0.176857 | 1.13674 | 0.186474 | 0.104783 | 3.942267 | A1 |
| 6.468395 | 0.183485 | 0.103067 | 0.040925 | 0.070475 | 0.007501 | 0.040005 | 0.022057 | 1.638072 | 0.029045 | 0.048903 | 3.062387 | 0.080385 | 0.020397 | 0.045031 | 0.009703 | 0.018189 | 0.620304 | 0.147035 | 1.028968 | 0.201045 | 0.093789 | 4.314593 | A2 |
| 6.743879 | 0.178459 | 0.130046 | 0.058913 | 0.075248 | 0.007063 | 0.031786 | 0.018792 | 1.541376 | 0.027067 | 0.048003 | 3.400372 | 0.084518 | 0.023004 | 0.042076 | 0.010572 | 0.014738 | 0.600879 | 0.176904 | 1.192759 | 0.165879 | 0.140479 | 4.176326 | A3 |
| 7.330284 | 0.173872 | 0.097938 | 0.040006 | 0.073707 | 0.007276 | 0.035802 | 0.021087 | 1.479013 | 0.026687 | 0.049037 | 3.307854 | 0.100186 | 0.023507 | 0.039979 | 0.008789 | 0.017077 | 0.583468 | 0.185987 | 1.103218 | 0.180759 | 0.100391 | 3.906985 | A4 |
| 6.804734 | 0.179035 | 0.110572 | 0.043057 | 0.071583 | 0.008005 | 0.036003 | 0.040078 | 1.525879 | 0.024078 | 0.046766 | 3.078563 | 0.088284 | 0.025507 | 0.044753 | 0.010752 | 0.014798 | 0.603578 | 0.179657 | 0.939578 | 0.187352 | 0.122453 | 3.776958 | A5 |
| 8.045783 | 0 | 0 | 0.111107 | 0.208586 | 0.010052 | 0.055078 | 0.043078 | 1.904787 | 0.040078 | 0.040789 | 2.043651 | 0.097894 | 0.013107 | 0.025379 | 0.014032 | 0.20574 | 0.521685 | 0.154903 | 1.56486 | 0.240544 | 0.156486 | 5.983652 | B2 |
| 8.408247 | 0 | 0 | 0.104573 | 0.210783 | 0.009174 | 0.050793 | 0.046769 | 2.110078 | 0.038789 | 0.044004 | 2.300472 | 0.111524 | 0.012315 | 0.030132 | 0.013659 | 0.022011 | 0.461579 | 0.165089 | 1.453152 | 0.210076 | 0.180136 | 5.747386 | B3 |
| 7.957369 | 0 | 0 | 0.089453 | 0.187352 | 0.009932 | 0.059793 | 0.043005 | 1.696759 | 0.041023 | 0.039769 | 1.950377 | 0.092018 | 0.014007 | 0.021787 | 0.017302 | 0.019837 | 0.502023 | 0.144737 | 1.394137 | 0.194768 | 0.129768 | 6.035279 | B4 |
| 8.108439 | 0 | 0 | 0.113067 | 0.230051 | 0.010803 | 0.055105 | 0.040513 | 1.908745 | 0.039463 | 0.041089 | 2.500754 | 0.117787 | 0.013221 | 0.023082 | 0.014415 | 0.023075 | 0.451362 | 0.174973 | 1.487980 | 0.210135 | 0.152769 | 5.597351 | B5 |
| 11.04835 | 0.005032 | 0.009376 | 0.076632 | 0.175319 | 0.004685 | 0.056324 | 0.038707 | 1.700073 | 0.053076 | 0.033047 | 2.674590 | 0.104394 | 0.015533 | 0.036073 | 0.025047 | 0.029796 | 0.550685 | 0.129794 | 2.073174 | 0.151426 | 0.216142 | 8.551583 | C1 |
| 10.48541 | 0.070013 | 0.013935 | 0.067352 | 0.190038 | 0.004197 | 0.060032 | 0.040807 | 1.659786 | 0.059004 | 0.036003 | 2.597868 | 0.086024 | 0.015089 | 0.0033003 | 0.021453 | 0.032462 | 0.504579 | 0.125075 | 2.276157 | 0.121078 | 0.249786 | 8.173525 | C2 |
| 10.83085 | 0.053026 | 0.015043 | 0.061006 | 0.200132 | 0.005002 | 0.056793 | 0.038879 | 1.590038 | 0.060785 | 0.032789 | 2.873251 | 0.082518 | 0.015507 | 0.039079 | 0.025563 | 0.030208 | 0.565023 | 0.131973 | 2.370769 | 0.149769 | 0.288759 | 8.839687 | C3 |
| 11.50062 | 0.004769 | 0.012107 | 0.070317 | 0.170348 | 0.004603 | 0.050989 | 0.042032 | 1.686573 | 0.053076 | 0.0033007 | 2.600346 | 0.099287 | 0.148010 | 0.030931 | 0.028023 | 0.029979 | 0.480062 | 0.114887 | 2.138031 | 0.180326 | 0.250537 | 8.403573 | C4 |
| 10.03349 | 0.049075 | 0.016352 | 0.073057 | 0.168319 | 0.004874 | 0.058032 | 0.040105 | 1.650035 | 0.055076 | 0.030782 | 2.660347 | 0.085489 | 0.016103 | 0.035073 | 0.026375 | 0.033045 | 0.500369 | 0.127893 | 2.507136 | 0.131124 | 0.231315 | 7.973654 | C5 |

**Table S4** By-product accumulation (including organic acids and amino acids) of LATR12 and LATR12-2*∆rpiB::ddh*_St_ in 5-L fermenters ^a^.

| *E. coli* strain | Organic acids (g/L) | | | | Amino acids (g/L) | | | | |
| --- | --- | --- | --- | --- | --- | --- | --- | --- | --- |
|  | PEP | Pyr | α-KG | OAA | Ala | Val | Leu | Met | Glu |
| LATR12 | 1.97 ± 0.08 | 4.16 ± 0.43 | 1.71 ± 0.13 | 1.44 ± 0.23 | 9.42 ± 0.73 | 7.64 ± 0.80 | 1.26 ± 0.14 | 0.51 ± 0.06 | 11.57 ± 1.07 |
| LATR12-2*∆rpiB::ddh*_St_ | 1.02 ± 0.11 | 2.73 ± 0.15 | 1.20 ± 0.19 | 0.79 ± 0.04 | 4.57 ± 0.21 | 3.18 ± 0.05 | 0.62 ± 0.05 | 0.16 ± 0.01 | 12.38 ± 0.95 |

^a^ PEP: Phosphoenolpyruvate, Pyr: Pyruvate; α-KG: α-ketoglutarate; OAA: Oxaloacetate; Ala: Alanine; Val: Valine; Leu: Leucine; Met: Methionine; Glu: Glutamate.

All data are meaning values of three determinations of three independent experiments with ± SD.

**Table S5** Overview on L-lysine producing strains of *Escherichia coli* and *Corynebacterium glutamicum*.

| Strain | Final titer  (g/L) | Carbon yield  (%) | Productivity  (g/(L∙h)) | Reference |
| --- | --- | --- | --- | --- |
| ***E. coli*** | | | | |
| LATR12-2*∆rpiB::ddh*_St_ | 119.5 | 49.1 | 2.99 | This work |
| 1^# a^ | 200~230 | 73~75 | 5.0~5.5 | Factory data in China |
| LATR11/pWG-*DC*^SM^*A*^SM^*BH*_c.g_*LP* | 125.6 | 59 | 3.14 | [^2^](#_ENREF_2) |
| NT1003 | 134.9 | 45.4 | 1.9 | [^7^](#_ENREF_7) |
| ZY0217 | 46.3 ^b^ | 27.8 ^b^ | 0.83 ^b^ | [^8^](#_ENREF_8) |
| WC196R/RSFP24 | 4.6 ^c^ | 0.2 ^d^ | 0.12 ^d^ | [^1^](#_ENREF_1) |
| ***C. glutamicum*** | | | | |
| 2^# a^ | 250~270 | 70~72 | 5.0~5.5 | Factory data in China |
| JL‑69P_tac‑M_ *gdh* | 181.5 | 64.6 | 3.78 | [^9^](#_ENREF_9) |
| LYS-12 | 120 | 55 | 4.0 | [^10^](#_ENREF_10) |
| JL-6 Δ*dapB::Ec-dapB*^C115G,G116C^ | 117.3 | 44 | 2.93 | [^11^](#_ENREF_11) |
| SEA-3 | 2.0 ^c^ | 0.2 ^d^ | 0.1 ^d^ | [^12^](#_ENREF_12) |

^a^ Used strain number due to the privacy concerns.

^b^ Used pretreated beet molasses as carbon source.

^c^ Achieved in shake‑flask fermentation.

^d^ Estimated from reference.

**Table S6** The plasmids used in this study

| Plasmids | Relevant characteristic(s) | Reference ^a^ | |
| --- | --- | --- | --- |
| pDXW-8 | Amp^r^ and Km^r^, *E. coli-C. glutamicum* shuttle vector with *tac* promoter | | [^13^](#_ENREF_13) |
| pET28a (+) | Km^r^, Expression vector with *T7* promoter 6×His tag and *lac* operator | | Stratagene |
| pKO3-Km | Integration vector | | Stratagene |
| pMD19-T/*ddh*_Cg_ | pMD19-T simple vector carrying *ddh* gene from *C. glutamicum* | | Chemosynthesis |
| pMD19-T/*ddh*_Bf_ | pMD19-T simple vector carrying *ddh* gene from *B. fragilis* | | Chemosynthesis |
| pMD19-T/*ddh*_Ct_ | pMD19-T simple vector carrying *ddh* gene from *C. therimocellum* | | Chemosynthesis |
| pMD19-T/*ddh*_Bs_ | pMD19-T simple vector carrying *ddh* gene from *B. sphaericus* | | Chemosynthesis |
| pMD19-T/*ddh*_St_ | pMD19-T simple vector carrying *ddh* gene from *S. thermophilum* | | Chemosynthesis |
| pMD19-T/*ddh*_Ut_ | pMD19-T simple vector carrying *ddh* gene from *U. thermosphaericus* | | Chemosynthesis |
| pET28a/*ddh*_Cg_ | pET28a (+) carrying *ddh* gene from *C. glutamicum* | | This work |
| pET28a/*ddh*_Bf_ | pET28a (+) carrying *ddh* gene from *B. fragilis* | | This work |
| pET28a/*ddh*_Ct_ | pET28a (+) carrying *ddh* gene from *C. therimocellum* | | This work |
| pET28a/*ddh*_Bs_ | pET28a (+) carrying *ddh* gene from *B. sphaericus* | | This work |
| pET28a/*ddh*_St_ | pET28a (+) carrying *ddh* gene from *S. thermophilum* | | This work |
| pET28a/*ddh*_Ut_ | pET28a (+) carrying *ddh* gene from *U. thermosphaericus* | | This work |
| pDXW-8/*ddh*_Cg_ | pDXW-8 carrying *ddh* gene from *C. glutamicum* | | This work |
| pDXW-8/*ddh*_Bf_ | pDXW-8 carrying *ddh* gene from *B. fragilis* | | This work |
| pDXW-8/*ddh*_Ct_ | pDXW-8 carrying *ddh* gene from *C. therimocellum* | | This work |
| pDXW-8/*ddh*_Bs_ | pDXW-8 carrying *ddh* gene from *B. sphaericus* | | This work |
| pDXW-8/*ddh*_St_ | pDXW-8 carrying *ddh* gene from *S. thermophilum* | | This work |
| pDXW-8/*ddh*_Ut_ | pDXW-8 carrying *ddh* gene from *U. thermosphaericus* | | This work |
| pKO3-Km/∆*dapD* | Integration vector for the knockout of the *dapD* gene | | This work |
| pKO3-Km/*dapD*^A1G^ | pKO3-Km carrying mutant *dapD* occurred *A→G* at genetic loci 1 | | This work |
| pKO3-Km/∆*rpiB* | Integration vector for the knockout of the *rpiB* gene | | This work |
| pKO3-Km/∆*dapD::ddh*_St_ | Integration vector for replacement of the *dapD* gene by the cassette of *ddh*_St_ gene | | This work |
| pKO3-Km/∆*rpiB::ddh*_Cg_ | Integration vector for replacement of the *rpiB* gene by the cassette of *ddh*_Cg_ gene | | This work |
| pKO3-Km/∆*rpiB::ddh*_St_ | Integration vector for replacement of the *rpiB* gene by the cassette of *ddh*_St_ gene | | This work |

^a^: The vectors pET28a (+) and pKO3-Km were purchased from BioVector NTCC Inc; The genes were chemo-synthesized by General Biosystems (Anhui), Inc. (Chuzhou, China).

**Table S7** The oligonucleotides used in this study

| Oligonucleotide ^a^ | | | 5’→3’ sequence ^b^ | Cleavage sites | | | Purposes ^c^ |
| --- | --- | --- | --- | --- | --- | --- | --- |
| *ddh*_Cg_-F | CTAGCTAGC**TAAGGAGGAAAAAAAA**ATGACCAACATCCGCGTAGC | | | | *Nhe*I | PCR for the *ddh*_Cg_ operon | |
| *ddh*_Cg_-R’ | GGGGTACCTTAGACGTCGCGTGCGATCAG | | | | *Kpn*I |  |  |
| *ddh*_Cg_-R’’ | CCGCTCGAGTTAGACGTCGCGTGCGATCAG | | | | *Xho*I |  |  |
| *ddh*_Bf_-F | CTAGCTAGC**TAAGGAGGAAAAAAAA**ATGAAAAAAGTAAGAGCAGC | | | | *Nhe*I | PCR for the *ddh*_Bf_ operon | |
| *ddh*_Bf_-R’ | GGGGTACCTTATACCAGGTGGCCGATCCATTC | | | | *Kpn*I |  |  |
| *ddh*_Bf_-R’’ | CCGCTCGAGTTATACCAGGTGGCCGATCCATTC | | | | *Xho*I |  |  |
| *ddh*_Ct_-F | CTAGCTAGC**TAAGGAGGAAAAAAAA***A*TGGAAAAGATAAGGATAG | | | | *Nhe*I | PCR for the *ddh*_Ct_ operon | |
| *ddh*_Ct_-R’ | GGGGTACCTTACAATAAACTTCTCCTG | | | | *Kpn*I |  |  |
| *ddh*_Ct_-R’’ | CCGCTCGAGTTACAATAAACTTCTCCTG | | | | *Xho*I |  |  |
| *ddh*_Bs_-F | CTAGCTAGC**TAAGGAGGAAAAAAAA**ATGAGTGCAATTCGAGTAG | | | | *Nhe*I | PCR for the *ddh*_Bs_ operon | |
| *ddh*_Bs_-R’ | GGGGTACCTTATAATAGTTCCTTACGTAATTG | | | | *Kpn*I |  |  |
| *ddh*_Bs_-R’’ | CCGCTCGAGTTATAATAGTTCCTTACGTAATTG | | | | *Xho*I |  |  |
| *ddh*_St_-F | CTAGCTAGC**TAAGGAGGAAAAAAAA**ATGGACAAGCTGCGCGTGGCG | | | | *Nhe*I | PCR for the *ddh*_St_ operon | |
| *ddh*_St_-R’ | GGGGTACCCTACACCAGCTTCCGGATCCACG | | | | *Kpn*I |  |  |
| *ddh*_St_-R’’ | CCGCTCGAGCTACACCAGCTTCCGGATCCACG | | | | *Xho*I |  |  |
| *ddh*_Ut_-F | CTAGCTAGC**TAAGGAGGAAAAAAAA**ATGAGTAAAATTAGAATTGGGATTG | | | | *Nhe*I | PCR for the *ddh*_Ut_ operon | |
| *ddh*_Ut_-R’ | GGGGTACCTTATAAAAGTTCTTTTCTTAAATC | | | | *Kpn*I |  |  |
| *ddh*_Ut_-R’’ | | CCCAAGCTTTTATAAAAGTTCTTTTCTTAAATC | | | *Hin*dIII | PCR for the *dapD* left arm, *dapD*-L | |
| *dapD-*L-F | | TCCCCCCGGGCGCATGGATAACATCGACGAAG | | | *Sal*I |  |  |
| *dapD-*L-R | | GAATGCGGCCGC***CGCGTTCCGGCGGGGTCAGTCGGCGAATTTCATCGGC***ACTTTG | | | *Not*I |  |  |
| *dapD*-R-F | | GAATGCGGCCGC***GCCGATGAAATTCGCCGACTCGCGTTCCGGCGGGGTC***TGTG | | | *Not*I | PCR for the *dapD* right arm, *dapD*-R | |
| *dapD-*R-R | | ACGCGTCGACCGCAAACCATACAAACTGC | | | *Sal*I |  |  |
| *rpiB*-L-F | | TCCCCCCGGGATCATCATGATGTGAGCATCAG | | | *Xma*I | PCR for the *rpiB* left arm, *rpiB*-L | |
| *rpiB-*L-R | | GAATGCGGCCGC***GATTGTGGATGCGTGGCTGACCACAAATCAAAATCCCG***CCATC | | | *Not*I |  |  |
| *rpiB*-R-F | | GAATGCGGCCGC***CGGGATTTTGATTTGTGGTGATTGTGGATGCGTGGCTG***GGCGC | | | *Not*I | PCR for the *rpiB* left arm, *rpiB*-L | |
| *rpiB-*R-R | | ACGCGTCGACAGGTCACGCCCCTGCCGCTC | | | *Sal*I |  |  |
| *P*_tac_-F | | GAATGCGGCCGCCCGTTCTGGATAATGTTTTTTGC | | | *Not*I | PCR for the *ddh*_X_ cassette with promoter *tac* and terminator *rrnBT1T2* | |
| *P*_tac_-R | | GAATGCGGCCGCGGGTTATTGTCTCATGAG | | | *Not*I |  |  |
| *lysC-*F^r^ | | GAACTGGCCGCGCTGCAGCTG | | | - | RT-PCR for *lysC* | |
| *lysC-*R^r^ | | CAGCAAGGCTGCCGTATAATC | | | - |  |  |
| *metL-*F^r^ | | CTGAAACGCGCGCAGGTACG | | | - | RT-PCR for *metL* | |
| *metL-*R^r^ | | TGACGCAGGCGCAGTTCG | | | - |  |  |

**Table S7** The oligonucleotides used in this study (Continued)

| Oligonucleotide ^a^ | | 5’→3’ sequence ^b^ | Cleavage sites | | | Purposes ^c^ |
| --- | --- | --- | --- | --- | --- | --- |
| *thrA-*F^r^ | CGTGGTAAATAACGATGATG | | | - | RT-PCR for *thrA* | |
| *thrA-*R^r^ | GCTGACGCTTCAGTTGCTC | | | - |  |  |
| *asd-*F^r^ | GTGCGCGACATATGCGTGAG | | | - | RT-PCR for *asd* | |
| *asd-*R^r^ | GCGCAGCTCACCGCTACG | | | - |  |  |
| *dapA-*F^r^ | CTATTTGTCGAACCCAATC | | | - | RT-PCR for *dapA* | |
| *dapA-*R^r^ | CATGCTTAAGCGCCGCTCTG | | | - |  |  |
| *dapB-*F^r^ | AGATTGCGCGGTCTACAGTC | | | - | RT-PCR for *dapB* | |
| *dapB-*R^r^ | CTCCAGACGCTCGCCAATATC | | | - |  |  |
| *dapD-*F^r^ | CGTATTAATGATAATCAG | | | - | RT-PCR for *dapD* | |
| *dapD-*R^r^ | CTGACGTACCGCCGCTGGTG | | | - |  |  |
| *dapC-*F^r^ | GATCACACCTGTGCGGTGGTG | | | - | RT-PCR for *dapC* | |
| *dapC-*R^r^ | CGCACTGCACTTCATCAAAC | | | - |  |  |
| *dapE-*F^r^ | ACAGGCGGAACGTCCGACG | | | - | RT-PCR for *dapE* | |
| *dapE-*R^r^ | CGTTGATACATACGGGCAAG | | | - |  |  |
| *dapF-*F^r^ | CGTGCCGTTTCGCGCTAAC | | | - | RT-PCR for *dapF* | |
| *dapF-*R^r^ | CAGGACCAAGCGAAAC | | | - |  |  |
| *ddh-*F^r^ | GACTCGATCATCCGGGC | | | - | RT-PCR for *ddh* | |
| *ddh-*R^r^ | CGTCATCGACAGCGCGTC | | | - |  |  |
| *lysA-*F^r^ | GCACGGTATCTGGTACACCG | | | - | RT-PCR for *lysA* | |
| *lysA-*R^r^ | TGACCTGACGCACCATAG | | | - |  |  |
| *lysP-*F^r^ | CACTTACGGTCAGAACTATG | | | - | RT-PCR for *lysP* | |
| *lysP-*R^r^ | CGCACTCCAGATCCAGCCCG | | | - |  |  |
| *amtB*-F^r^ | CGCGATTGCCGGTCTGGTCG | | | - | RT-PCR for *amtB* | |
| *amtB*-R^r^ | GTGCACACCGAAGACATCGC | | | - |  |  |
| *glnD*-F^r^ | CATATCGATACTTTCCAGCG | | | - | RT-PCR for *glnD* | |
| *glnD*-R^r^ | GTCGGCAATCTGGCTGAATC | | | - |  |  |
| 16S-F^r^ | GCTCGTGTTGTGAAATGTTG | | | - | RT-PCR for 16S rRNA | |
| 16S-R^r^ | GTAAGGGCCATGATGACTTG | | | - |  |  |

^a^ For two reverse primers, one (i.e., *X*-R’) was used to construct the recombinant pDXW-8, and the other (i.e., *X*-R’’) was used to construct the recombinant pET-28a; ^r^: The premiers for RT-PCR.

^b^ Cleavage sites are underlined; Nucleotide in bold and italic: Homologous sequences; Nucleotide in bold: Shine-Dalgarno (SD) sequences [^14^](#_ENREF_14); Sequence in italic: Mutation base.

^c^ The *ddh*_X_ indicates the *ddh* gene from different bacteria.

-: No cleavage sites.

**Fig. S1.** Alignment of nucleotide (a) and amino acids (b) sequences of DapDHs from different bacteria. Identical sequences are presented in same underpainting. The ddhCg (or CgDapDH), ddhBs (or BsDapDH), ddhCt (or CtDapDH), ddhBf (or BfDapDH), ddhSt (or StDapDH) and ddhUt (or UtDapDH) represent the *ddh* gene or DapDH from *C. glutamicum* ATCC13032, *B. sphaericus* IFO3525, *C. therimocellum* ATCC27405, *B. fragilis* YCH46, *S. thermophilum* IAM14863 and *U. thermosphaericus* A1, respectively. CgDHDPR and MtDHDPR represent the dihydrodipicolinate reductase from *C. glutamicum* and *Mycobacterium tuberculosis*, respectively.


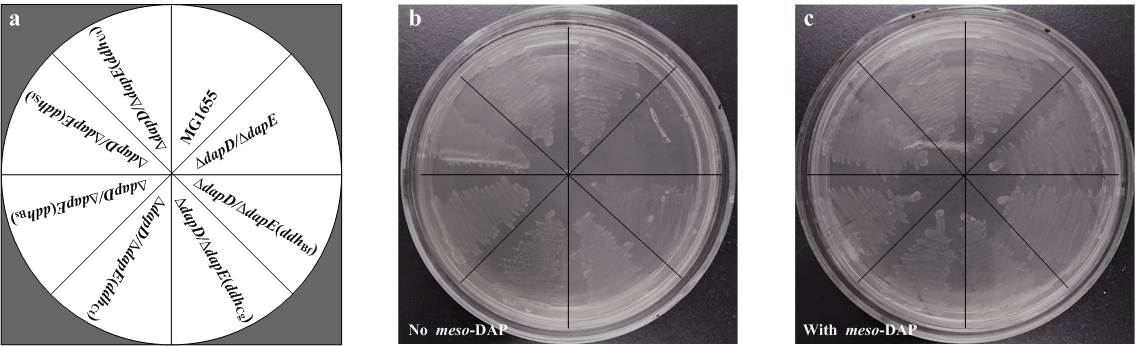


**Fig. S2.** Functional complementation of the *E. coli* Δ*dapD*/Δ*dapE* double mutant during cultivating on MS medium without *meso*-DAP (b) or with *meso*-DAP (c) at 40℃ for 10 h. (a) is shown the mutant code, and MG1655 represents *E. coli* MG1655.


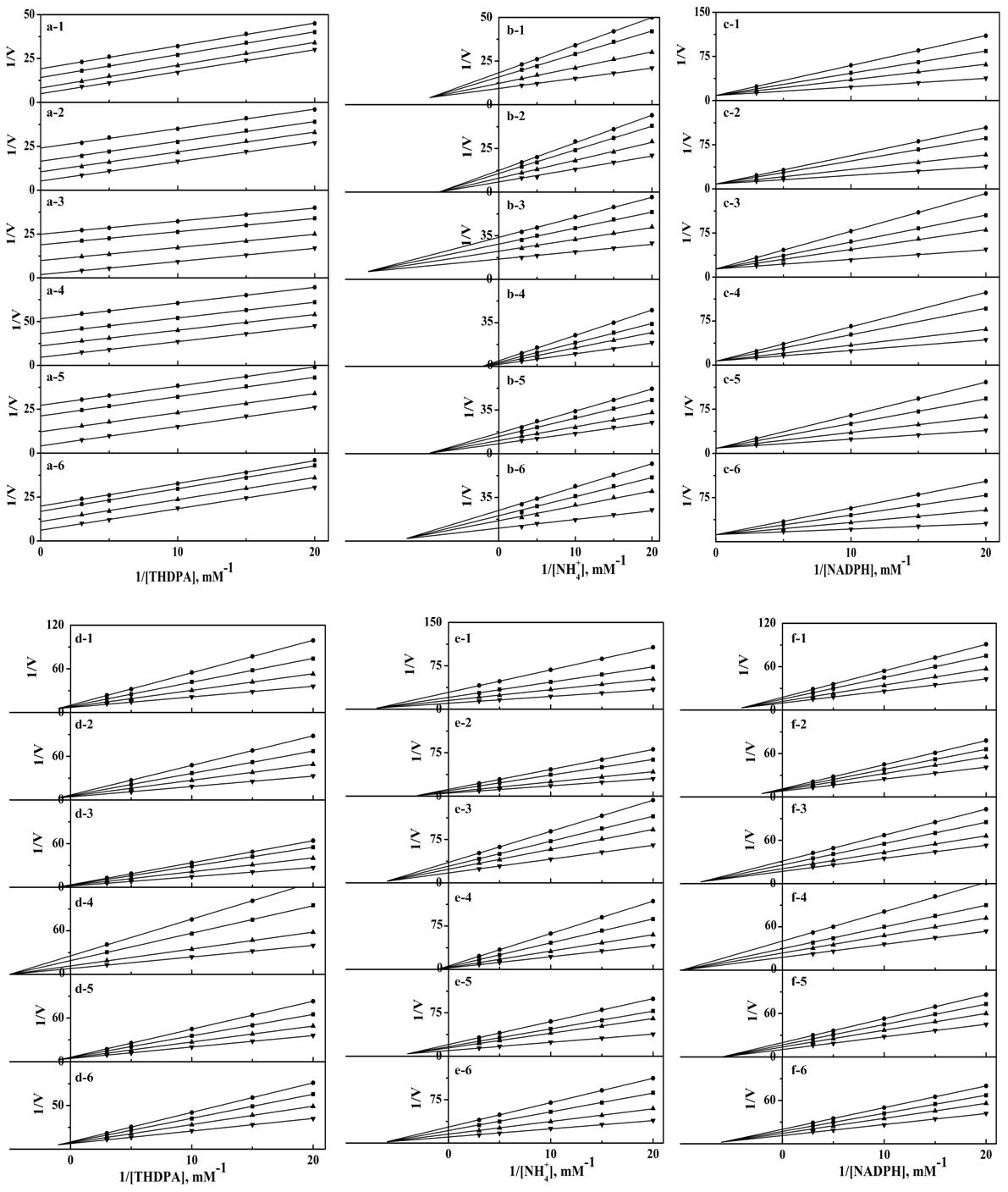


**Fig. S3.** Product inhibition with THDPA, $\mathrm{NH}_{4}^{+}$ or NADPH as the varied substrate. (a) Product inhibition by NADP^+^ with THDPA as the varied substrate.$\mathrm{NH}_{4}^{+}$ and NADPH were held at 200 mmol/L and 0.3 mmol/L, respectively. The concentration of NADP^+^ used were 0 μmol/L (●), 100 μmol/L (■), 150 μmol/L (▲), 200 μmol/L (▼). (b) Product inhibition by NADP^+^ with $\mathrm{NH}_{4}^{+}$ as the varied substrate. THDPA and NADPH were held at 5 mmol/L and 0.3 mmol/L, respectively. The concentration of NADP^+^ used were 0 μmol/L (●), 100 μmol/L (■), 150 μmol/L (▲), 200 μmol/L (▼). (c) Product inhibition by NADP^+^ with NADPH as the varied substrate.$\mathrm{NH}_{4}^{+}$ and THDPA were held at 200 mmol/L and 5 mmol/L, respectively. The concentration of NADP^+^ used were 0 μmol/L (●), 50 μmol/L (■), 100 μmol/L (▲), 150 μmol/L (▼). (d) Product inhibition by *meso*-DAP with THDPA as the varied substrate.$\mathrm{NH}_{4}^{+}$ and NADPH were held at 200 mmol/L and 0.3 mmol/L, respectively. The concentration of *meso*-DAP used were 0 μmol/L (●), 100 μmol/L (■), 150 μmol/L (▲), 200 μmol/L (▼). (e) Product inhibition by *meso*-DAP with THDPA as the varied substrate. THDPA and NADPH were held at 5 mmol/L and 0.3 mmol/L, respectively. The concentration of *meso*-DAP used were 0 μmol/L (●), 100 μmol/L (■), 200 μmol/L (▲), 300 μmol/L (▼). (f) Product inhibition by *meso*-DAP with THDPA as the varied substrate.$\mathrm{NH}_{4}^{+}$ and THDPA were held at 200 mmol/L and 5 mmol/L, respectively. The concentration of *meso*-DAP used were 0 μmol/L (●), 100 μmol/L (■), 200 μmol/L (▲), 300 μmol/L (▼).

**Fig. S4.** Flask fermentation of *E. coli* LATR12 under different temperature. (a) Growth curves (dotted lines) and residual glucose (solid lines); (b) L-lysine accumulation (black bars) and productivity (gray bars). Signal denotes: 30℃ (■), 33℃ (◆), 37℃ (▲), 40℃ (**×**), 45℃ (●), and 50℃ (**+**). The data represent mean values and standard deviations obtained from three independent cultivations.

**Fig. S5.** The cell growth (a) and L-lysine production (b) of strains LATR12 (black bars) and LATR12∆*rpiB* (gray bars) during cultivating on MS medium. The data represent mean values and standard deviations obtained from three independent cultivations.

**Fig. S6.** Analyzing the expression levels of *amtB* and *glnD* from LATR12*∆dapD::ddh*_St_, LATR12*∆rpiB::ddh*_St_ and LATR12-2*∆rpiB::ddh*_St_ by semiquantitative RT-PCR during growth on MS medium without (NH_4_)_2_SO_4_ and with 20 g/L of (NH_4_)_2_SO_4_. The numerical value in Figures represents fold change of gene expression level between different culture conditions.

**Supplementary References**

1 Imaizumi, A. *et al.* Improved production of L-lysine by disruption of stationary phase-specific *rmf* gene in *Escherichia coli*. *J Biotechnol* **117**, 111-118, doi:10.1016/j.jbiotec.2004.12.014 (2005).

2 Xu, J. Z., Han, M., Ren, X. D. & Zhang, W. G. Modification of aspartokinase III and dihydrodipicolinate synthetase increases the production of L-lysine in *Escherichia coli*. *Biochem Eng J* **114**, 82-89, doi:10.1016/j.bej.2016.06.025 (2016).

3 Link, A. J., Phillips, D. & Church, G. M. Methods for generating precise deletions and insertions in the genome of wild-type *Escherichia coli*: Application to open reading frame characterization. *J Bacteriol* **179**, 6228-6237, doi:DOI 10.1128/jb.179.20.6228-6237.1997 (1997).

4 Trigoso, Y. D., Evans, R. C., Karsten, W. E. & Chooback, L. Cloning, Expression, and purification of histidine-tagged *Escherichia coli* dihydrodipicolinate reductase. *PloS One* **11**, e0146525, doi:10.1371/journal.pone.0146525 (2016).

5 Hudson, A. O. *et al.* Dual diaminopimelate biosynthesis pathways in *Bacteroides fragilis* and *Clostridium thermocellum*. *Biochim Biophys Acta* **1814**, 1162-1168, doi:10.1016/j.bbapap.2011.04.019 (2011).

6 Schnell, R. *et al.* Tetrahydrodipicolinate *N*-succinyltransferase and dihydrodipicolinate synthase from *Pseudomonas aeruginosa*: Structure analysis and gene deletion. *PloS One* **7**, doi:10.1371/journal.pone.0031133 (2012).

7 Ying, H. X., He, X., Li, Y., Chen, K. Q. & Ouyang, P. K. Optimization of culture conditions for enhanced lysine production using engineered *Escherichia coli*. *Appl Biochem Biotech* **172**, 3835-3843, doi:10.1007/s12010-014-0820-7 (2014).

8 He, X. *et al.* Enhanced L-lysine production from pretreated beet molasses by engineered *Escherichia coli* in fed-batch fermentation. *Bioproc and Biosys Eng* **38**, 1615-1622, doi:10.1007/s00449-015-1403-x (2015).

9 Xu, J. Z., Wu, Z. H., Gao, S. J. & Zhang, W. G. Rational modification of tricarboxylic acid cycle for improving L-lysine production in *Corynebacterium glutamicum*. *Microb Cell Fact* **17**, 105-118 (2018).

10 Becker, J., Zelder, O., Hafner, S., Schroder, H. & Wittmann, C. From zero to hero-Design-based systems metabolic engineering of *Corynebacterium glutamicum* for L-lysine production. *Metab Eng* **13**, 159-168, doi:10.1016/j.ymben.2011.01.003 (2011).

11 Xu, J. Z., Yang, H. K., Liu, L. M., Wang, Y. Y. & Zhang, W. G. Rational modification of *Corynebacterium glutamicum* dihydrodipicolinate reductase to switch the nucleotide-cofactor specificity for increasing L-lysine production. *Biotechnol Bioeng* **115**, 1764-1777, doi:10.1002/bit.26591 (2018).

12 Hoffmann, S. L. *et al.* Lysine production from the sugar alcohol mannitol: Design of the cell factory *Corynebacterium glutamicum* SEA-3 through integrated analysis and engineering of metabolic pathway fluxes. *Metab Eng* **47**, 475-487, doi:10.1016/j.ymben.2018.04.019 (2018).

13 Xu, D. Q., Tan, Y. Z., Shi, F. & Wang, X. Y. An improved shuttle vector constructed for metabolic engineering research in *Corynebacterium glutamicum*. *Plasmid* **64**, 85-91, doi:10.1016/j.plasmid.2010.05.004 (2010).

14 Ringquist, S. *et al.* Translation initiation in *Escherichia coli* - Sequences within the ribosome-binding site. *Mol Microbiol* **6**, 1219-1229, doi:DOI 10.1111/j.1365-2958.1992.tb01561.x (1992).
